# Activation of ROP6 GTPase by Phosphatidylglycerol in Arabidopsis

**DOI:** 10.3389/fpls.2018.00347

**Published:** 2018-03-15

**Authors:** Xiuli Han, Yue Shi, Guoyong Liu, Yan Guo, Yongqing Yang

**Affiliations:** State Key Laboratory of Plant Physiology and Biochemistry, College of Biological Sciences, China Agricultural University, Beijing, China

**Keywords:** GTPase, ROP6, Phosphatidylglycerol, *Arabidopsis*, Activity

## Abstract

Plant Rho-like GTPases (ROPs) are switch-like proteins which play essential roles in controlling cell polarity development and cellular activities. ROPs are regulated by many factors, such as auxin, light, and RopGEFs and RopGAPs proteins. However, it has not been reported yet whether small molecules play a role in the regulation of ROP activity. Here, we showed that AtROP6 specially bound to a phospholipid, phosphatidylglycerol (PG), by the protein-lipid overlay and liposome sedimentation assays, and further MST assay gave a dissociation constant (Kd) of 4.8 ± 0.4 μM for binding of PG to His-AtROP6. PG profile analysis in Arabidopsis revealed that PG existed both in leaves and roots but with distinctive fatty acyl chain patterns. By evaluating AtROP6 activity using RIC1 effector binding-based assay, we found that PG stimulated AtROP6 activity. In the FM4-64 uptake experiment, PG inhibited AtROP6-mediated endocytosis process. By evaluating internalization of PIN2, PG was shown to regulate endocytosis process coordinately with NAA. Further root gravitropism experiment revealed that PG enhanced the AtROP6-mediated root gravity response. These results suggest that the phospholipid PG physically binds AtROP6, stimulates its activity and influences AtROP6-mediated root gravity response in Arabidopsis.

## Introduction

Rho-related GTPases from plants (ROPs), switch-like molecules between its GTP-binding active state and GDP-binding inactive state, play essential roles in controlling cell polarity development and cellular activities (Li et al., 1998; Craddock et al., 2012), and are involved in cellular vesicular trafficking, cytoskeleton activities, cell-shape formation, root-hair development and pollen tube growth (Craddock et al., 2012). ROPs in Arabidopsis contain 11 ROPs family members from ROP1 to ROP11, wherein, AtROP2, AtROP4 and AtROP6 are all involved in polar cell growth and cell polarity, but AtROP6 functions antagonistically to AtROP2 and AtROP4 in the generation of jigsaw-shaped pavement cells (Craddock et al., 2012). In plant cells, the ROP6-RIC1 signaling pathway participates in cortical microtubule ordering and cell expansion to keep jigsaw-puzzle appearance of pavement cells (Fu et al., 2009), and further study reveals that auxin acts upstream of AtROP6 and AtROP2 to control this process (Xu et al., 2010). The ROP6-RIC1 signaling pathway is also involved in cortical microtubule arrangement via RIC1 physically interacting with microtubule severing protein katanin (KTN1) and activating its severing activity (Lin et al., 2013). In Arabidopsis roots and etiolated hypocotyls, auxin induces re-orientation of microtubule from transverse to longitudinal to inhibit cell expansion via ROP6-RIC1-KTN1 signaling pathway (Chen et al., 2014). The ROP6-RIC1 signaling pathway negatively regulates clathrin-mediated endocytosis and inhibits BFA-sensitive PIN-FORMED 1 (PIN1) and PIN2 auxin transporters’ internalization to influence auxin-mediated root gravistimulation response and leaf vein pattern in Arabidopsis (Chen et al., 2012). SPIKE1, a DOCK family protein encoding a guanine nucleotide exchange factor in Arabidopsis, acts upstream of ROP6-RIC1 signaling pathway to maintain subcellular PIN2 polar distribution via inhibition of PIN internalization (Basu et al., 2008; Lin D. et al., 2012). SPIKE1 is also reported to activate AtROP2, AtROP4 and AtROP6 to modulate anisotropic growth and shape in Arabidopsis petal (Ren et al., 2016). ROP6 is also involved in cell development, pathogen response, F-actin bundle formation and symbiotic fungus growth in Arabidopsis (Poraty-Gavra et al., 2013; Venus and Oelmuller, 2013), and nodule formation in *Lotus japonicas* (Ke et al., 2012; Wang et al., 2015a,b).

Lipids, a class of amphoteric compounds in organisms comprising fatty acids, such as glycerolipids, glycerophospholipids, sphingolipids, sterol lipids, saccharolipids (Lam and Shui, 2013), play essential roles in cellular structures as well as cellular activities as signaling molecules. Phosphatidylinositol 4, 5-bisphosphate (PIP2), a phospholipid principally localized in the plasma membrane, functions as a necessary cofactor in the modulation of many ion channels and transporters in plasma membrane, including transient receptor potential (TRP) channel, voltage-gated K^+^ channel superfamily, voltage-gated Ca^2+^ channel, voltage-gated Na^+^ channel, inward-rectifier K^+^ channel, Ca^2+^ release channel, two-P domain K^+^ channel, and ion transporters such as the Na^+^/H^+^ antiporter (Suh and Hille, 2008). KCNQ channel, a type of voltage-gated K^+^ channel, shows increased affinity to PIP_2_ when its arginine residues are methylated by methyltransferase, which further leads to seizure suppression in mice (Kim et al., 2016). Phosphatidic acid (PA) is considered as a second messenger in plants, which responses to many biotic and abiotic stresses such as wounding, plant defense and oxidative stress, osmotic stress, abscisic acid (ABA) treatment, ethylene treatment and Nod factor treatment (Munnik, 2001). In Arabidopsis, PA binds to MAP65-1 to regulate microtubule organization in response to salt stress (Zhang et al., 2012). In yeast, PA is considered as a pH biosensor that links membrane biogenesis to metabolism (Young et al., 2010). In mammalian cells, PA content is increased with mitogenic stimulation, and then interacts with the domain in mTOR (mammalian target of rapamycin) to activate mTOR downstream effectors (Fang et al., 2001). Phosphatidylserine (PS) was reported to play active roles in enteroviral infection, for PS-enriched vesicles are more efficient in viral infection than single viral particles and PS is a co-factor for enteroviral infection in subsequent infectivity and transmission (Chen et al., 2015). Eicosapolyenoic acids in Arabidopsis are involved in ABA-mediated drought response; transgenic plants with higher eicosapolyenoic acids content are more sensitive to ABA and exogenous application of eicosapolyenoic acids can mimic ABA-mediated drought response (Yuan et al., 2014). Oleic acid (18:1) in Arabidopsis is involved in defense response by physically interacting with NOA1, leading to its degradation and regulating NO synthesis (Mandal et al., 2012). In Arabidopsis, sphingosine-1-phosphate (S1P) is reported to be a signaling molecule regulating ABA-mediated stomatal apertures and guard cell ion channel activities via heterotrimeric G proteins downstream elements (Ng et al., 2001; Coursol et al., 2003).

In Arabidopsis, AtROP6 is activated via association with lipid rafts by palmitic (C16:0) or stearic (C18:0) acids transient S-acylation in its cysteines (Sorek et al., 2010). As lipids in the plasma membrane are involved in regulating many membrane proteins’ function (Munnik, 2001; Tejos et al., 2014), whether lipids are involved in AtROP6 regulation has never been reported. In this study, a phospholipid phosphatidylglycerol (PG) was identified to bind to AtROP6 in protein-lipid overlay assay, liposome sedimentation assay and microscale thermophoresis (MST) assay. PG was found both in roots and leaves with distinctive fatty acyl chain patterns. Exogenous application of PG activated AtROP6 activity, inhibited AtROP6-mediated endocytosis process, enhanced root gravitropic response, and regulated endocytosis process coordinately with 1-naphthylacetic acid (NAA). Thus, we suggest that PG physically binds to AtROP6, regulates its activity, and further influences AtROP6-mediated seedling polarity development.

## Materials and Methods

### Chemicals

PC (phosphatidylcholine, catalog number 850375), PE (phosphatidylethanolamine, catalog number 850725), PG (phosphatidylglycerol, catalog number 841148), PA (phosphatidic acid, catalog number 840875), and DG (diglyceride, catalog number 800811) were purchased from Avanti Polar Lipids, Inc.; PI (phosphatidylinositol), PS (phosphatidylserine), DGDG (digalactosyldiacylglycerol), MGDG (monogalactosyldiacylglycerol), and SQDG (sulfoquinovosyl diacylglycerol) were purchased from Lipid Products, United Kingdom. LPA (lysophosphatidic acid, catalog number L7260), MG (monoglyceride, catalog number M7765), and NAA (1-naphthylacetic acid) were ordered from Sigma-Aldrich. FM4-64 was ordered from Molecular Probes, Inc.

### Plant Material and Growth Conditions

All *Arabidopsis thaliana* lines used in this study are as follows: Columbia-0 (Col-0), 35S::GFP-ROP6 was kindly provided by Dr. Ying Fu (China Agricultural University), *PIN2-GFP*, *rop6^CA^* and *rop6-2* were kindly provided by Dr. Deshu Lin (Fujian Agriculture and Forestry University). Arabidopsis seeds were sterilized, sown on 0.443% (w/v) Murashige and Skoog salts (MS, Sigma-Aldrich) medium plus 20 g/L sucrose (pH 5.8), and grown vertically or horizontally in controlled growth chamber at 22°C under 16-h light/8-h dark cycle for 5–10 days (light intensity of 50 μmol m^-2^s^-1^) unless indicated otherwise. For soil growth, the seedlings were then transferred to soil under a 16-h light (22°C)/8-h dark (20°C) cycle.

### Cloning, Expression, and Purification of AtROP6 Construct and Purification of MBP-RIC1 Construct

The coding sequence of *AtROP6* was amplified with the R6-Bf/R6-Hr primer and cloned into the pET-30a vector between the *BamH*I and *Hind* III sites to generate the recombinant plasmid pET-30a-AtROP6. The coding sequences of *AtROP1* and *AtROP3* were amplified with the R1-Ef/R1-Sr, R3-Ef/R3-Sr primers and cloned into the pGEX-6p1 vector between the *EcoR*I and *Sal*I sites to generate the recombinant plasmid 6p1-AtROP1 and 6p1-AtROP3, respectively. The coding sequence of *AtROP6* was amplified with the R6-Bf/R6-Er primer and cloned into the pGEX-6p1 vector between the *BamH*I and *EcoR*I sites to generate the recombinant plasmid 6p1-AtROP6. The coding sequence of *14-3-3λ* was amplified with the λ-Bf/λ-Sr primer and cloned into the pGEX-6p1 vector between the *BamH*I and *Sal*I sites to generate the recombinant plasmid 6p1-14-3-3λ. Mutation of Thr-20 to Asn was generated by first amplification with the R6TN-f/R6-Er, R6-Bf/R6TN-r primers and second amplification with the R6-Bf/R6-Er primers, and then cloned into the pGEX-6p1 vector to generate the recombinant plasmid 6p1-AtROP6^DN^, or cloned into the pCAMBIA1390-GFP vector to generate the recombinant plasmid 1390-GFP-AtROP6^DN^. Mutation of Gly-15 to Val was generated by first amplification with the R6GV-f/R6-Er, R6-Bf/R6GV-r primers and second amplification with the R6-Bf/R6-Er primers, and then cloned into the pGEX-6p1 vector to generate the recombinant plasmid 6p1-AtROP6^CA^, or cloned into the pCAMBIA1390-GFP vector to generate the recombinant plasmid 1390-GFP-AtROP6^CA^. The plasmid was verified by sequencing and was then transformed into the bacterial strain BL21 or protoplast, respectively. Primer sequences are listed in Supplementary Table 1.

Bacterial cells were grown in Luria-Bertani medium supplemented with 50 mg/L kanamycin or 100 mg/L ampicillin at 37°C until cells reached an optical density OD_600 nm_ from 0.7 to 0.9. The recombinant protein was expressed at 37°overnight after induction with 0.4 mM of IPTG. Proteins were purified using Ni-beads as described in the manufacturer’s instructions.

The bacterial strain expressing MBP-RIC1 in pMAL21 construct was kindly provided by Dr. Ying Fu (China Agricultural University). MBP-RIC1-Conjugated Amylose Beads was prepared as described previously (Xu, 2012).

### Lipid-Protein Overlay Assay

Stocks of phospholipids were first prepared in organic solvents according to the manufacturer’s instructions or in the lipid-soluble solvent: DCM:MeOH:H_2_O 65:35:8 (v/v/v). Lipid-protein overlay assay was performed as described previously (Sun et al., 2013). Briefly, lipid test strip was prepared by spotting the indicated lipids with the amount of 5 nmol onto a PVDF membrane and kept dry for 1 h at room temperature. The lipid test strip was incubated with 1 μg/mL of His fusion ROP6 protein in 3% BSA-20 mM Tris-HCl (pH 8.0) for 2 h at room temperature for blocking and incubation. After washing with PBST (0.1% tween 20) buffer, the presence of bound ROP6 protein was detected using mouse anti-His-tag antibody as the primary antibody and goat anti-mouse antibody conjugated to HRP as the secondary antibody.

### Liposome Sedimentation Assay

Stocks of phospholipids were first prepared in organic solvents according to the manufacturer’s instructions or in the lipid-soluble solvent: DCM:MeOH:H_2_O 65:35:8 (v/v/v). Liposome sedimentation assay was performed as described previously (Sun et al., 2013). Briefly, liposome mixture was prepared with a weight ratio of 1:1 DOPC/DOPE and a serial weight ratio of PG. The total lipid weight was 200 μg. The solvent in the mixtures was first removed using nitrogen gas for 30 min without heating and kept dry in a desiccator overnight at room temperature. The lipids were then hydrated in 200 μL buffer containing 100 mM NaCl, 1 mM NaN_3_ and 20 mM Tris-HCl (pH 6.8) at 67°C for 1 h, during which time the lipids were mixed by pipetting up and down with a micropipette every 15 min. After being freeze-thawed three times, the liposomes were formed by sonication. The liposomes were further pelleted, re-suspended in 40 μL buffer containing 100 mM NaCl, 1 mM NaN_3_ and 20 mM Tris-HCl (pH 6.8), and protein (10 μg) was then added. Incubation was performed with the liposome for 30 min at room temperature. Centrifugation at 12,000 rpm gets liposome-bound pellet fraction and free-protein supernatant fraction, which were further analyzed by SDS-PAGE and Coomassie Blue Staining. Quantitation of protein content was performed with ImageJ software.

### Microscale Thermophoresis (MST) Assay

MST assay was carried out using Monolith NT.115 instrument (NanoTemper Technologies) as described previously (Lin C.C. et al., 2012; Entzian and Schubert, 2016). The buffer with Tris salt in the purified recombinant proteins was first replaced with PBST buffer (0.005% tween 20, pH = 7.5) using column A supplied by the manufacturer. Then the proteins at a final concentration of 5 μM were labeled with excess NHS NT-647 dye at a molar ratio of 1:5 at room temperature for 30 min in the dark according to the manufacturer’s instructions. Free unlabeled dye was removed using column B pre-equilibrated with PBST buffer (0.005% tween 20, pH = 7.5). PG (1 mg) was first dissolved in 50 μL organic solvent DCM:MeOH:H_2_O 65:35:8 (v/v/v), and then dried with nitrogen gas for 30 min and kept dry in a desiccator overnight at room temperature. The dried PG was dissolved in 1 mL PBST buffer (0.005% tween 20, pH = 7.5) by hydration for 1 h at room temperature until no lipid could be seen on the Eppendorf tube wall, and was then centrifuged at 12,000 rpm for 10 min at room temperature to remove the possible existing pellet. The PG solution was serially diluted with PBST buffer and mixed with the same amount of labeled protein. The samples were loaded into capillaries (NanoTemper Technologies) and analyzed. The assay was carried out with 20% LED power and 20% MST power. Signal Thermophoresis + T-Jump Data were used for calculating dissociation constant (Kd). Data was analyzed using software NT Analysis and Origin9.

### Chemical Treatment

FM4-64 was dissolved in distilled water, BFA and cycloheximide (CHX) were dissolved in dimethyl sulfoxide, and NAA was dissolved in methanol. For phenotypic analysis, PG was dissolved in methanol. For other experiments, the solvent to dissolve PG was indicated in the experiment.

### ROP6 Activity Assay

ROP6 activity assay was performed as described previously (Xu, 2012). ROP6 with activity could be pulled down by MBP-RIC1-conjugated agarose beads, while ROP6 without activity could not be, so MBP-RIC1-conjugated agarose beads and GFP-tagged ROP6 were both need to be prepared.

For preparation of MBP-RIC1-conjugated agarose beads, MBP-RIC in pMAL21 construct was transformed into *Escherichia coli*. Bacterial cells BL21 and the cells were cultured in Luria-Bertani medium with 100 mg/L ampicillin at 37°C. When the optical density of the cells reached about 0.8, the cells were cooled to 16°C and 0.4 mM of IPTG was added into the medium. The expression of MBP-RIC fusion protein was induced overnight at 16°C and then purified according to the manufacturer’s instructions.

For preparation of GFP-tagged ROP6, 10-day-old 35S::GFP-ROP6 transgenic seedlings were treated with indicated amounts of PG or without PG in liquid 1/2 MS for 48 h, and then the seedlings were grounded with liquid nitrogen and extracted at 4°C for 1 h in extraction buffer: 25 mM Hepes, 10 mM MgCl_2_, 1 mM EDTA, 100 mM KCl, 5 mM DTT, 5 mM Na_3_VO_4_, 5 mM NaF, 1 mM PMSF, 1% Triton X-100, pH 7.4, in which, DTT, Na_3_VO_4_, PMSF were added before use. After extraction, 50 μL of the total protein was transferred out for the final total protein analysis, and for the reserved solution, the centrifugation of 10,000 *g* at 4°C was used to remove the debris and the same volume of extraction buffer without Triton X-100 was added into the total protein in the supernatant.

For preparation of GFP-tagged ROP6^*CA*^ and GFP-tagged ROP6^*DN*^, the plasmids were purified by CsCl gradient centrifugation. Then the Col-0 protoplasts were prepared and the plasmids were transformed into the protoplasts as described previously (Sheen, 2001). The GFP-tagged ROP6^*CA*^ and GFP-tagged ROP6^*DN*^ were prepared as described previously (Xu, 2012).

Then the MBP-RIC1-conjugated agarose beads were added into the supernatant and incubated at 4°C for 3 h. After incubation, the beads were washed five times with washing buffer: 25 mM Hepes, 5 mM MgCl_2_, 1 mM EDTA, 1 mM DTT. The MBP-RIC1 bound GFP-ROP6 was separated by SDS-PAGE and detected using mouse anti-GFP antibody as the primary antibody and goat anti-mouse antibody conjugated to HRP as the secondary antibody.

### FM4-64 Staining and PIN2 Internalization Observation

FM4-64 staining was performed as described previously (Chen et al., 2012). Three-day-old seedlings were transferred to MS medium supplemented with or without indicated amounts of PG. After growing for 48 h, the seedlings were first labeled with 2 μM FM 4-64 for 5 min, washed out for three times, and further incubated in liquid 1/2 MS medium for 20 min at room temperature. PIN2 internalization observation was performed as described previously (Chen et al., 2012). Three-day-old seedlings were transferred to MS medium supplemented with or without indicated amounts of PG. After growing for 48 h, the seedlings were treated with 50 μM BFA or 50 μM BFA plus 5 μM NAA for 2 h. For confocal microscopy observation, the seedlings were mounted on glass slides in 10% glycerol for observation under confocal microscope (Andor Dradonfly spinning disk confocal, Nikon TiE microscope, plan APO 60×, NA1.4 objective, and andor zyla4.2plus sCMOS camera) with excitation wavelength 561 nm and emission wavelength 620–650 nm for FM4-64 observation and excitation wavelength 488 nm and emission wavelength 507 nm for GFP observation. Quantitation of the fluorescence signal was performed with ImageJ software.

### Root Gravity Response Assay

Five-day-old seedlings were transferred to the indicated amounts of PG-containing MS medium and applied recovered growth for 12 h, and were then gravity stimulated for 12 h (at 90° rotation). The root tips were labeled at indicated time periods and the angles formed were measured using ImageJ software.

### PG Content Analysis

Quantitation of PG content in Arabidopsis was performed as described previously with minor modification (Milne et al., 2005; Hsu et al., 2007). Five-day-old seedlings were transferred to MS medium supplemented with or without 0.1 μM NAA. After growing for 5 days, the root part and leaf part were collected separately using a scissor, and only 2/3 root part near the root tip was collected to avoid hypocotyl contamination.

The samples were grounded under liquid nitrogen, and 200 μL ice-cold 2:1 CH_2_Cl_2_:CH_3_OH (containing 0.25% 12 M HCl, v/v) was added into each sample. After a vortex for 2 min, 40 μL of 1 M HCl was added and vortexed for 30 s. Then the samples were centrifuged for 5 min at 12,000 *g* at 4°C, and the lower organic phase was transferred to a new tube and dried using a vacuum centrifuge (Eppendorf Concentrator plus). The extracted lipids were re-dissolved in 100 μL CH_2_Cl_2_-CH_3_OH-H_2_O-300 mM piperidine (1:1:0.2:0.1, v/v/v/v) for further HRESIMS (high-resolution electrospray ionization-mass spectrometry) analysis (Thermo Fisher, Q-Exactive).

## Results

### PG Binds to AtROP6

AtROP6, a plasma membrane-localized protein in Arabidopsis, functions as a molecular switch in many cellular signaling responses (Sorek et al., 2010; Craddock et al., 2012). To investigate whether lipids in the plasma membrane are involved in the regulation of AtROP6 function, we performed a protein-lipid overlay screen assay to look for the possible lipids that AtROP6 physically interacts with.

**FIGURE 1:**
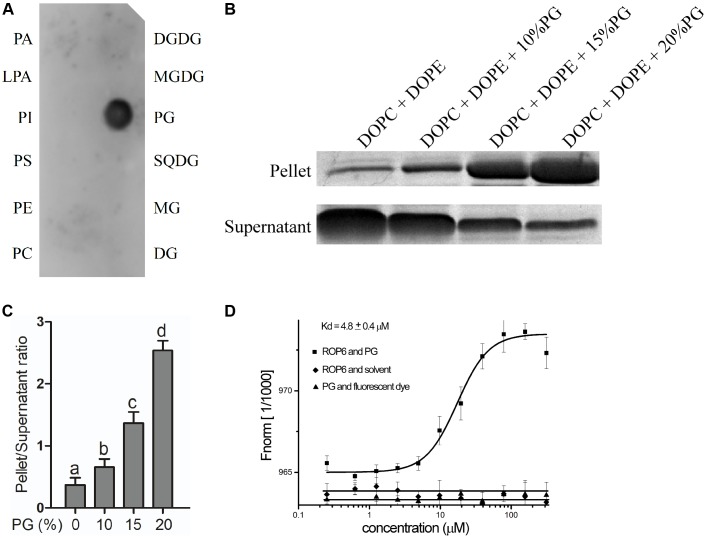
PG binds to AtROP6. **(A)** Lipid-protein overlay screen assay between the recombinant protein His-AtROP6 and the lipids. His-AtROP6 was extracted and purified from *E. coli*. The amount of each lipid spot is 5 nmol. PA, Phosphatidic acid; LPA, Lysophosphatidic acid; PI, Phosphatidylinositol; PS, Phosphatidylserine; PE, phosphatidylethanolamine; PC, phosphatidylcholine; DGDG, digalactosyldiacylglycerol; MGDG, monogalactosyldiacylglycerol; PG, phosphatidylglycerol; SQDG, sulfoquinovosyl diacylglycerol; MG, monoglyceride, DG, diglyceride. **(B)** Liposome sedimentation assay between the recombinant protein His-AtROP6 and liposomes with or without PG. Liposome mixtures with a total lipid amount of 200 μg were prepared by a weight ratio of 1:1 DOPC/DOPE and a serial weight ratio of PG. The amount of PG that each liposome contained is shown on the top. Liposomes were incubated with protein His-AtROP6 and centrifuged to get liposome-bound pellet protein fraction and supernatant protein fraction. Further detection use SDS-PAGE and Coomassie Blue Staining. **(C)** Ratio quantitation analysis of His-AtROP6 content in **(B)**. The protein content in the supernatant and the pellet was performed quantitation separately using ImageJ software, and then the ratio was calculated. **(D)** Microscale thermophoresis assay between the recombinant protein His-AtROP6 and PG. His-AtROP6 was labeled with NHS NT-647 dye and kept at a constant concentration (100 nM). PG was hydrated in 1 mL PBST buffer (0.005% tween 20, pH = 7.5) to get the stock solution 1 mg/mL. PG was titrated from 30 nM to 300 μM and the assay was carried out with 20% LED power and 20% MST power. The binding between His-AtROP6 and PG was fitted and the affinity was calculated as 4.8 ± 0.4 μM. The negative controls are the bindings between His-AtROP6 and PBST buffer solvent, and between NHS NT-647 dye and PG, which all have no binding and could not be fitted. The bar represents the mean and the error bar represents the standard error. The data was calculated from at least three independent experiments. The statistical significance was analyzed by a Student’s *t*-test and the significant differences (*P* ≤ 0.05) are indicated by lowercase letters.

Glycerophospholipids including PA, PI, PS, PE, LPA, PG, PC and glycerolipids including MG, DG, MGDG, DGDG, SQDG are lipids that were reported to be located or possibly located at the plasma membrane (Manoharan et al., 1985; Ritter and Yopp, 1993; Drøbak et al., 1994; Moreau et al., 1998; Andersson et al., 2003; Kim et al., 2004; Jouhet et al., 2007; Li et al., 2007, 2016; Yang et al., 2010; Dong et al., 2012). We spotted these lipids onto the PVDF membrane and performed the binding assay between these lipids and AtROP6. The recombinant protein His-ROP6 was purified from *E. coli* strain BL21 expressing recombinant plasmid pET-30a-AtROP6. The result showed that His-AtROP6 specially bound to PG, but not other lipids (**Figure 1A**). Further interaction assay between PG and His-casein kinase1-like protein2 (CKL2) (Zhao et al., 2016), a negative control, revealed that PG specially bound to His-AtROP6, not His-CKL2 (Supplementary Figure 1A).

To further verify the interaction between PG and AtROP6, we performed a liposome sedimentation assay. We prepared control liposome containing only PC and PE, and also prepared PG-containing liposomes. PG-containing liposomes were prepared with the same total lipid content as the control liposome by replacing equal amounts of PC and PE with PG. After incubation with the recombinant protein His-AtROP6, the liposomes were separated into the pellet and the supernatant by centrifugation, in which liposome-bound His-AtROP6 existed in the pellet and liposome-unbound His-AtROP6 existed in the supernatant. After analysis by SDS-PAGE and Coomassie Blue Staining, we found that the protein level of His-AtROP6 increased dramatically in the pellet with the elevated PG content in the liposomes (**Figures 1B,C**). This result supports the evidence that AtROP6 specially binds to PG, not PC and PE.

We also performed MST assay to investigate the binding affinity between AtROP6 and PG. To prepare the PG solution, we used PBST (0.005% tween 20) to hydrate PG to get the protein-compatible PG solution. His-AtROP6 was labeled with fluorescent dye and mixed with a series dilution of PG. The dissociation constant (Kd) between His-AtROP6 and PG was determined to be 4.8 ± 0.4 μM, while PG and fluorescent dye, His-AtROP6 and solvent (PBST, 0.005% tween 20) had no interaction and could not be fitted (**Figure 1D**). These results suggest that AtROP6 specially binds to PG, and the dissociation constant is 4.8 ± 0.4 μM.

To further investigate whether PG binds to other ROP protein family members, we performed liposome sedimentation assay and MST assay between PG and GST-AtROP1, GST-AtROP3 or GST-AtROP6. In the liposome sedimentation assay, after liposome preparation and protein incubation, we found that the protein level of GST-AtROP1, GST-AtROP3 and GST-AtROP6 increased in the pellet with the elevated PG content (Supplementary Figure 1B). By contrast, the negative control protein levels of GST-tag and GST-14-3-3λ in the pellet did not change with the increase of PG content in the liposomes (Supplementary Figure 1C). In the MST assay, the recombinant proteins were labeled with fluorescent dye and mixed with a series dilution of PG. The binding affinities between PG and GST-AtROP1, GST-AtROP3, or GST-AtROP6 were calculated as 22.4 ± 0.7 μM, 34.5 ± 0.9 μM, or 11.6 ± 0.3 μM (Supplementary Figure 1D). The negative controls between GST tag and PG, and GST-14-3-3λ and PG had no interaction (Supplementary Figure 1D). These results indicate that other ROP protein family members AtROP1 and AtROP3 also bind to PG but with a little weaker interaction compared with AtROP6. The differences in the binding affinity between His-ROP6 and PG, and GST-ROP6 and PG might due to the influence of tag on ROP6 structure. We also performed the interaction test between PG and AtROP6^CA^ (mutation of Gly-15 to Val, a constitutively active form of ROP6) or AtROP6^DN^ (mutation of Thr-20 to Asn, a dominant negative form of ROP6), which showed that both GST-AtROP6^CA^ and GST-AtROP6^DN^ bound to PG in the liposome sedimentation assay (Supplementary Figure 2).

These results suggest that PG not only binds to AtROP6, but also binds to other ROP protein family members, such as AtROP1 and AtROP3.

### PG Activates AtROP6 Activity

**FIGURE 2:**
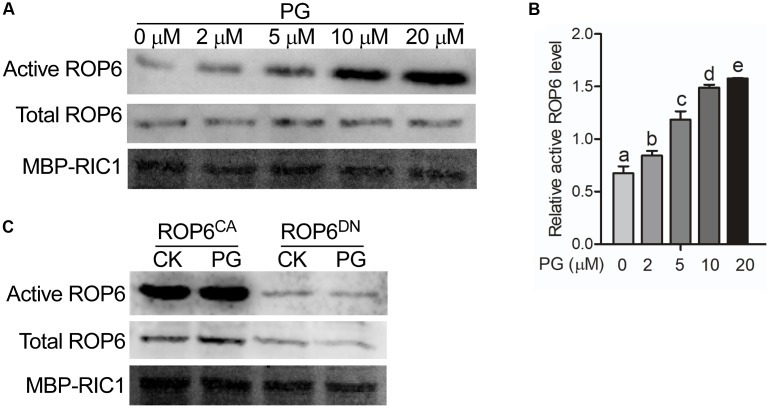
PG activates AtROP6 in a dose-dependent manner. **(A)** Immunoblot analysis of active ROP6 and total ROP6 from *35S::GFP-ROP6* seedlings treated with indicated amounts of PG. The upper lane shows the immunoblot analysis of active ROP6, which was from MBP-RIC1 bound fraction. The middle lane shows the immunoblot analysis of total ROP6, which was prepared from total protein extraction. ROP6 was detected by immunoblot using anti-GFP antibody. The lower lane shows the Coomassie Brilliant Blue staining of MBP-RIC1 on PVDF membrane after immunoblot analysis. **(B)** Relative active ROP6 level in **(A)**. The amount of active ROP6 and total ROP6 was performed quantitation separately using ImageJ software, and then the amount of active ROP6 was divided by the amount of total ROP6 to get the relative active ROP6 level. **(C)** Immunoblot analysis of active ROP6 and total ROP6 from *35S::GFP-ROP6^CA^* and *35S::GFP-ROP6^DN^* seedlings treated with 20 μM PG. The upper lane shows the immunoblot analysis of active ROP6. The middle lane shows the immunoblot analysis of total ROP6. ROP6 was detected by immunoblot using anti-GFP antibody. The lower lane shows the Coomassie Brilliant Blue staining of MBP-RIC1 on PVDF membrane after immunoblot analysis. The bar represents the mean and the error bar represents the standard error. The data was calculated from at least three independent experiments. The statistical significance was analyzed by a Student’s *t*-test and the significant differences (*P* ≤ 0.05) are indicated by lowercase letters.

PG is widely studied as a thylakoid lipid in plants, which plays essential roles in oxygenic photosynthesis (Babiychuk et al., 2003; Kobayashi et al., 2016). PG is also reported to localize in the oat root with decreased content when plant is under phosphate-limitation condition (Andersson et al., 2003). However, the function of PG in the root has never been reported. In our study, we found that PG binds to AtROP6. To investigate what role PG binding to AtROP6 plays, we performed and AtRop6 activity assay. ROP6 activity could be analyzed by an effector binding-based assay using RIC1, an effector of AtROP6, which specifically binds to the active form of ROP6 but not to the inactive form of ROP6 (Xu, 2012). To test the effect of PG on ROP6 activity, Arabidopsis seedlings expressing *35S::GFP-ROP6* were treated with indicated amounts of PG, followed by a pulled-down assay using MBP-RIC1-conjugated agarose beads and active ROP6 was detected with anti-GFP antibody. As shown in **Figures 2A,B**, we found that ROP6 activity was increased with the addition of PG and the activity displayed a dose-dependent manner with the increase of PG concentration. PC and PE were also applied on the seedlings expressing *35S::GFP-ROP6*; however, no stimulation of ROP6 activity was seen (Supplementary Figure 3). In the interaction assay, PG also bound to AtROP6^CA^ and AtROP6^DN^, so we further performed a ROP6 activity assay both in the protoplasts expressing *35S::GFP-ROP6*^CA^ and protoplasts expressing *35S::GFP-ROP6*^DN^, and no stimulation effect was seen in either of them after PG treatment (**Figure 2C**). These results suggest that the interaction between PG and AtROP6 plays a role in stimulating AtROP6 activity.

### PG Exists Both in Arabidopsis Leaves and Roots but With Distinctive Fatty Acyl Chain Patterns

**FIGURE 3:**
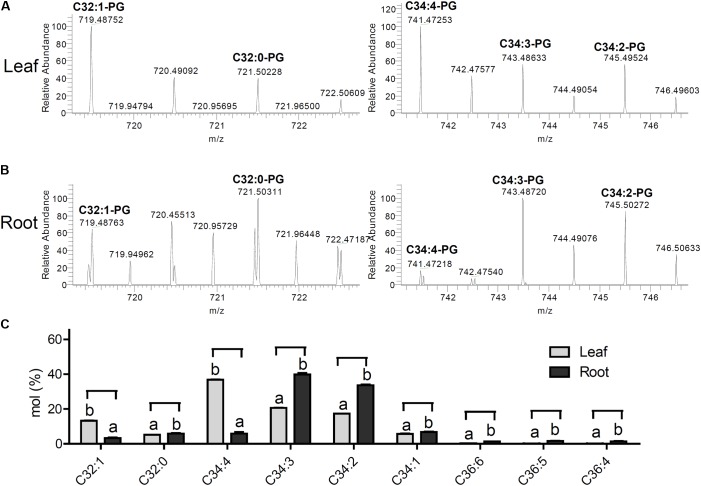
PG exists both in leaves and roots with distinctive fatty acyl patterns. **(A)** The high-resolution mass spectrum of PGs from Arabidopsis leaves ([M-H]^-^, ESI^-^). 719.48752 C32:1-PG (calcd. for C_38_H_72_O_10_P: 719.48631); m/z 721.50228 C32:0-PG (calcd. for C_38_H_74_O_10_P: 721.50196); m/z 741.47253 C34:4-PG (calcd. for C_40_H_70_O_10_P: 741.47066); m/z 743.48633 C34:3-PG (calcd. for C_40_H_72_O_10_P:743.48631); m/z 745.49524 C34:2-PG (calcd. for C_40_H_74_O_10_P: 745.50196). **(B)** The high-resolution mass spectrum of PGs from Arabidopsis roots ([M-H]^-^, ESI^-^). 719.48763 C32:1-PG (calcd. for C_38_H_72_O_10_P: 719.48631); m/z 721.50311 C32:0-PG (calcd. for C_38_H_74_O_10_P: 721.50196); m/z 741.47218 C34:4-PG (calcd. for C_40_H_70_O_10_P: 741.47066); m/z 743.48720 C34:3-PG (calcd. for C_40_H_72_O_10_P:743.48631); m/z 745.50272 C34:2-PG (calcd. for C_40_H_74_O_10_P: 745.50196). **(C)** PG composition analysis in the lipid extracts of Arabidopsis leaves and roots. The bar represents the mean and the error bar represents the standard error. The data were calculated from three independent experiments. The statistical significance was analyzed by a Student’s t-test and the significant differences (*P* ≤ 0.05) are indicated by lowercase letters.

Since PG activates AtROP6 activity, we want to investigate the distribution of PG species in Arabidopsis. The lipids from Arabidopsis leaves and roots were extracted separately, and then analyzed by mass spectrometry. By evaluating PG species, we found that PG existed both in leaves and roots but with distinctive fatty acyl chain patterns (**Figures 3A–C**). The PG from leaves showed nine species with the major species being C34:4-PG, which is similar to the work previously reported (Hsu et al., 2007). However, the PG from roots showed the major species being C34:3-PG. Besides C34:3-PG and C34:4-PG, all other PG species showed dramatic difference between roots and leaves (**Figures 3A–C**). These results suggest that PG exists in Arabidopsis but with distinctive fatty acyl chain patterns between roots and leaves.

### PG Is Involved in AtROP6-Mediated Endocytosis Regulation

**FIGURE 4:**
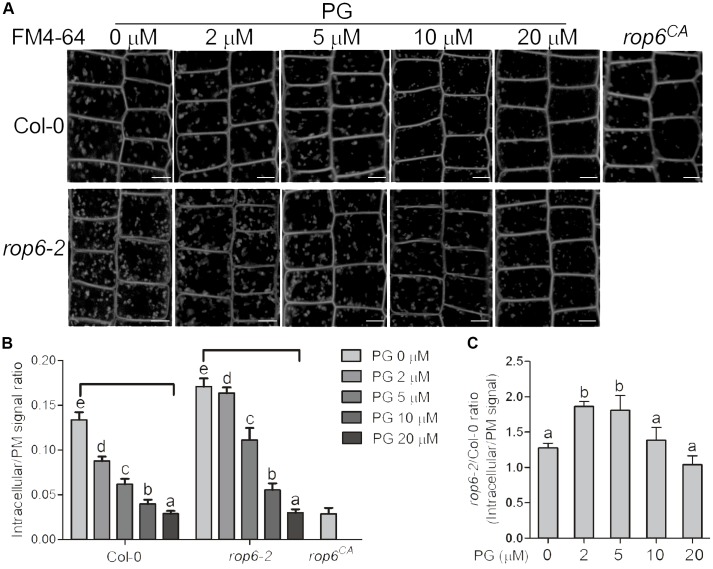
PG positively regulates ROP6-mediated endocytosis regulation. **(A)** FM4-64 uptake in the seedlings of Col-0, *rop6-2* and *rop6*^CA^. Seedlings of Col-0 and *rop6-2* were treated with indicated amounts of PG, and then stained with FM4-64 to observe endocytosis process. The amount of PG that each treatment used was shown on the top. The upper lane shows the FM4-64 uptake in the seedlings of Col-0. The lower lane shows the FM4-64 uptake in the seedlings of *rop6-2. rop6*^CA^ was stained with FM4-64 and observed for endocytosis process. Scale bar represents 5 μm. **(B)** Ratio quantitation analysis of FM4-64 signal in **(A)** in bar graph. The FM4-64 signal in the cytoplasm and the plasma membrane was performed measurement separately using ImageJ software, and then the ratio was calculated (*n* > 20). **(C)** Ratio quantitation analysis of FM4-64 signal between Col-0 and *rop6-2* in **(B)**. The bar represents the mean and the error bar represents the standard error. The data were calculated from at least three independent experiments. The statistical significance was analyzed by a Student’s t-test and the significant differences (*P* ≤ 0.05) are indicated by lowercase letters.

AtROP6 was reported to function in membrane trafficking in plants (Chen et al., 2012). Amphiphilic styryl dye FM4-64 is a marker widely used to monitor endocytosis in plants, which stains plasma membrane and is integrated into vesicles accompanied by endocytosis process (Rigal et al., 2015). As AtROP6 regulates endocytosis in Arabidopsis in previous report (Chen et al., 2012), and PG bound to and activated AtROP6 in our study, we performed an endocytosis experiment to investigate whether PG is involved in this process. Arabidopsis seedlings of Col-0 were first applied treatment with indicated amounts of PG or solvent, and then the root was stained with FM4-64 and endocytosis was observed. We found that, with PG treatment, the uptake of FM4-64 was reduced in wildtype Col-0 in a dose-dependent manner (**Figures 4A,B**), which is consistent with the PG concentration on ROP6 activity. When PG was applied at 20 μM, the endocytosis process in Col-0 seedlings was almost completely inhibited, which is similar to that in *rop6*^CA^ (a constitutively active mutant of ROP6) (**Figures 4A,B**). When we also applied treatment with PC and PE on the seedlings of Col-0, no inhibition of FM4-64 uptake was seen (Supplementary Figures 4A,B). To further investigate whether PG inhibits endocytosis process through ROP6, we applied the same PG treatment on the *rop6-2* mutant, which had been reported as a knockout mutant of ROP6 and showed increased endocytosis process in previous report (Chen et al., 2012). The endocytosis process in *rop6-2* also displayed a dose-dependent inhibition with PG treatment and showed complete inhibition at 20 μM (**Figures 4A,B**); however, the endocytosis process in *rop6-2* showed an obvious slower rate in the reduction of FM4-64 uptake compared with Col-0 at lower PG concentrations of 2 and 5 μM (**Figure 4C**). These results indicate that the endocytosis process in plants could be inhibited by PG, and the inhibition is partially through ROP6.

### PG Regulates Endocytosis Process Coordinately With Auxin

**FIGURE 5:**
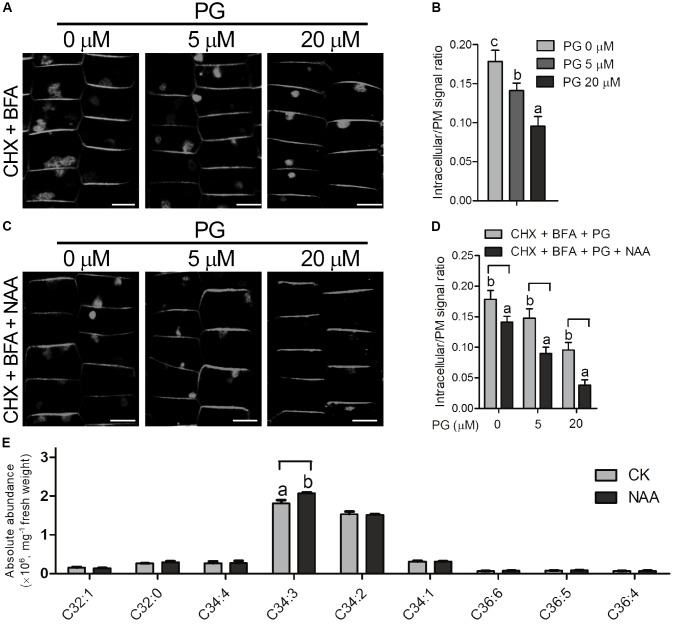
PG regulates endocytosis process coordinately with auxin. **(A)** The seedlings (*PIN2-GFP* in Col-0 background) were treated with indicated amounts of PG for 2 days, followed by treatment with CHX and indicated amounts of PG for 60 min, and then by treatment with CHX, BFA and indicated amounts of PG for 2 h. The concentration of the chemicals used: CHX, 50 μM; BFA, 50 μM. The amount of PG that each treatment used was shown on the top. Scale bar represents 5 μm. **(B)** Ratio quantitation analysis of PIN2-GFP signal in **(A)**. The PIN2-GFP signal in the cytoplasm and the plasma membrane was measured separately using ImageJ software, and then the ratio was calculated (*n* > 20). **(C)** BFA-induced PIN2 internalization was more inhibited by PG and NAA. The seedlings (*PIN2-GFP* in Col-0 background) were treated with indicated amounts of PG for 2 days, followed by treatment with CHX and indicated amounts of PG for 30 min, and by treatment with CHX, NAA and indicated amounts of PG for another 30 min, and then by treatment with CHX, NAA, BFA and indicated amounts of PG for 2 h. The concentration of the chemicals used: CHX, 50 μM; BFA, 50 μM, NAA 5 μM. The amount of PG that each treatment used was shown on the top. Scale bar represents 5 μm. **(D)** Ratio quantitation analysis of PIN2-GFP signal in **(A)**. PG content analysis in the lipid extracts of Arabidopsis roots with or without NAA treatment **(E)**. The PIN2-GFP signal in the cytoplasm and the plasma membrane was performed measurement separately using ImageJ software, and then the ratio was calculated (*n* > 20). The bar represents the mean and the error bar represents the standard error. The data were calculated from at least three independent experiments. The statistical significance was analyzed by a Student’s *t*-test and the significant differences (*P* ≤ 0.05) are indicated by lowercase letters.

Auxin inhibits endocytosis process in plants, thereby internalization of PIN-FORMED (PIN) proteins of auxin transporters is inhibited and further auxin efflux is promoted (Paciorek et al., 2005; Chen et al., 2012). As ROP6 is required for auxin inhibition of endocytosis in the previous report (Chen et al., 2012), and PG is involved in AtROP6-mediated endocytosis regulation in our study, we applied NAA treatment to investigate whether PG is involved in the auxin inhibition of endocytosis process. Brefeldin A (BFA) is a fungal toxin repressing the endosomal recycling of plasma membrane proteins in plants, which could induce PIN protein aggregation and form BFA bodies in the cytoplasm (Irani and Russinova, 2009; Chen et al., 2012). We first applied BFA treatment on the seedlings of Arabidopsis Col-0 in PIN2-GFP background to investigate the internalization of PIN2 signals, which showed BFA-induced PIN2 aggregates in the cytoplasm of epidermal cells (**Figures 5A,B**). To exclude the possibility that the accumulation of BFA bodies came from *de novo* PIN2-GFP synthesis, we applied a protein synthesis inhibitor CHX to investigate this process. CHX blocks translational elongation step in protein synthesis and is widely used as a protein synthesis inhibitor (Paciorek et al., 2005; Pan et al., 2009; Wang et al., 2016). Consistent with previous report (Pan et al., 2009), PIN2-GFP accumulation in BFA bodies was still observed in the presence of CHX (Supplementary Figures 5A,B). When PG was applied on the treatment, the accumulation of BFA bodies was decreased (**Figures 5A,B**), which is consistent with the PG inhibition on the uptake of FM4-64. When we applied treatment with PC and PE on the seedlings, no inhibition of BFA bodies was seen (Supplementary Figures 6A,B). Then we performed this BFA-induced PIN2 internalization experiment with NAA treatment, which showed that auxin inhibits PIN2 internalization as previously reported (Paciorek et al., 2005; Pan et al., 2009; Chen et al., 2012) however, PIN2-GFP accumulation in the BFA bodies upon NAA treatment (**Figures 5C,D**) was much less than that in the mock treatment (**Figures 5A,B**) in the seedlings pre-treated with PG, indicating that PG enhances NAA effect on the inhibition of endocytosis. To investigate whether PG affects exocytosis, we performed a BFA washout experiment to restore PIN2-GFP signals at the plasma membrane, which showed that the BFA bodies have no significant difference in all the seedlings with or without PG treatment (Supplementary Figures 7A,B). This indicates that PG inhibits endocytosis process, but not exocytosis process.

To investigate whether the content of PG changes with NAA treatment, we performed PG content analysis from Arabidopsis roots with or without NAA treatment using mass spectrometry. With 0.1 μM of NAA treatment, C34:3-PG, the major species of PG in the root, increased with NAA treatment, and the other PG species showed no significant difference compared with seedlings without NAA treatment (**Figure 5E**).

### PG/ROP6 Signaling Pathway Is Involved in AtROP6-Mediated Root Gravity Response

**FIGURE 6:**
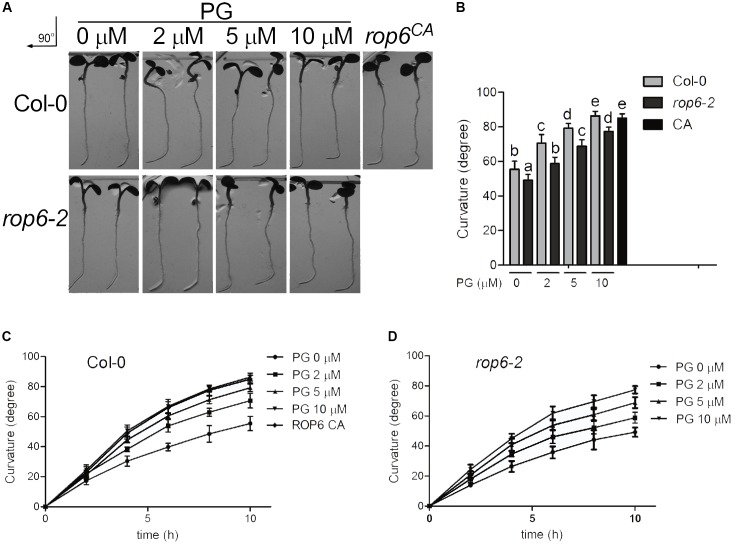
PG positively regulates ROP6-mediated gravitropic response. **(A)** Gravitropic response in the seedlings of Col-0, *rop6-2* and *rop6*^CA^. Seedlings of Col-0 and *rop6-2* were treated with indicated amounts of PG for 10 h, and then rotated 90° for gravistimulation. The amount of PG that each treatment used was shown on the top. The upper lane shows the gravitropic response in the seedlings of Col-0. The lower lane shows the gravitropic response in the seedlings of *rop6-2. rop6*^CA^ was rotated 90° for gravistimulation. **(B)** Root tropic bending curvatures of Col-0 and *rop6-2* at 10 h after re-orientation. **(C)** Root tropic bending curvatures of Col-0 after re-orientation at intervals of 2 h. **(D)** Root tropic bending curvatures of *rop6-2* after re-orientation at intervals of 2 h. The bar represents the mean and the error bar represents the standard error. The data were calculated from at least three independent experiments. The statistical significance was analyzed by a Student’s *t*-test and the significant differences (*P* ≤ 0.05) are indicated by lowercase letters.

It was reported that ROP6 pathway acts downstream of auxin in the regulation of endocytosis process in Arabidopsis roots (Chen et al., 2012). Root gravity response in plants is an auxin-mediated developmental process beneficial for plant adaption to its environment and ROP6 is required for this seedling development (Chen et al., 2012). Previous reports showed that the roots in *rop6*^CA^ exhibit a hypergravitropic response, whereas display an attenuated gravitropic response in loss-of-function mutant *rop6-2* (Chen et al., 2012; Lin D. et al., 2012). To determine whether PG regulates root gravity response in Arabidopsis, seedlings of Col-0 were applied treatment with indicated amounts of PG or solvent, and reoriented by 90° for gravity stimulation. We found that root gravitropic bending curvatures increased with the treatment of PG in a dose-dependent manner at all time points after gravity stimulation (**Figures 6A–C**), which is consistent with the PG concentration on the ROP6 activity and endocytosis phenotype except that the treatment of PG at 20 μM did not show regular pattern on root gravity response and 50 μM showed a waved pattern on root growth (Supplementary Figure 8). As negative control, PC and PE did not change root gravitropic bending curvatures in Col-0 seedlings (Supplementary Figures 9A,B). As root gravity response is dependent on root growth, we applied PG treatment to investigate whether PG influences root growth. After measuring the root length of the seedlings, we found that PG did not enhance root growth (Supplementary Figures 10A,B). We further investigated the PG effect on the *rop6-2* mutant seedlings, which also exhibited an increase in gravitropic bending curvatures after seedling re-orientation, but was much less compared with Col-0 seedlings at PG concentrations of 2 and 5 μM (**Figures 6A,B,D**), suggesting that PG elevates gravity response partially through ROP6. Taken together, these results indicate that PG regulates the root gravity response in Arabidopsis and this regulation is partially through ROP6.

## Discussion

Lipids play essential roles in cellular activities and mainly function as building blocks for the membrane structure, signaling molecules in the signal transduction, and membrane lipid environment for the function of membrane proteins (Bogdanov et al., 2009; Bogdanov et al., 2014; Dowhan, 2017). Many qualitative assays of the protein-lipid interaction have been developed and reported. Nuclear magnetic resonance (NMR) is an accurate and widely used technique; however, NMR is limited to the high requirement for proteins in the molecular weight, purity and stability. Liposome sedimentation assay is an acknowledged method to study lipid-protein interactions, with liposome being prepared and target proteins being precipitated on the liposome (Baron and Malhotra, 2002; Sun et al., 2013). Isothermal titration calorimetry (ITC) (Swamy and Sankhala, 2013), surface plasmon resonance (SPR) (Del and Stahelin, 2016), and MST (Dijkman and Watts, 2015) are quantitative techniques widely applied to study lipid-protein interactions and could afford binding constant. The protein-lipid overlay (PLO) assay is also a widely used technique to study lipid–protein interactions (Baron and Malhotra, 2002; Dowler et al., 2002; Munnik and Wierzchowiecka, 2013; Sun et al., 2013). Here, we applied a PLO screen assay to investigate the lipids that might interact with AtROP6, a plasma membrane switch-like molecule in Arabidopsis, functioning in cell polarity development and cellular activities. We found that AtROP6 specially binds to PG, not other lipids in our PLO screen assay. Further liposome sedimentation assay and MST assay verified the binding between PG and AtROP6. In our AtROP6 activity assay, we found that PG could activate AtROP6, inhibit FM4-64 uptake in the membrane trafficking, and regulate root gravity response through ROP6. Taken together, our attempt to study lipid-protein interaction let us find a phospholipid, PG, binds and regulates ROP6 activity in cellular activities.

Endocytic pathways in plants have been identified as clathrin-mediated endocytosis and membrane microdomain-associated endocytosis (Fan et al., 2015). Previous studies show that ROP6 regulates clathrin-mediated endocytosis (Chen et al., 2012; Nagawa et al., 2012; Wang et al., 2015a,b), and some other ROP protein family members are reported to be involved in membrane trafficking (Bloch et al., 2005; Lee et al., 2008; Nagawa et al., 2012). Since PG also bound to AtROP1 and AtROP3 in our study (Supplementary Figures 1B,D) and many components exist in the endocytosis process (Paciorek et al., 2005; Chen X. et al., 2011; Wang et al., 2013, 2016; Fan et al., 2015), it is possible that either other ROP protein family members or other components in the endocytic pathway are involved in this process to inhibit endocytosis process coordinately with AtROP6, which need further experiment to investigate it.

In the root gravitropism experiment, PG elevated gravity response both in Col-0 seedlings and *rop6-2* seedlings, but the *rop6-2* seedlings showed less sensitivity to PG compared with Col-0 seedlings, which further indicate that PG regulates cellular activities partially through AtROP6, and AtROP6 is not the only target protein of PG. In view of the former studies, many lipids have more than one protein target, such as phosphoinositides in Arabidopsis, which have many protein targets including PH-domain containing proteins, FYVE-domain containing proteins and PX-domain containing proteins (Lemmon, 2003; Leeuwen et al., 2004; Simon et al., 2014; Heilmann, 2016a,b). Phosphatidic acid in Arabidopsis was also reported to have many protein targets, such as ABI1 phosphatase 2C (Zhang et al., 2004), MAP65-1 (Zhang et al., 2012), and MPK6 (Yu et al., 2010). Because the target proteins of PG were studied less in Arabidopsis, further study would be possible to look for the other target proteins of PG.

In plants, PG was most widely studied as building block of thylakoid membrane, which plays crucial roles for oligomerization of photosystem I reaction center, electron transfer both in donor and acceptor sides of photosystem II, and the loss of PG biosynthesis in Arabidopsis has impaired oxygenic photosynthesis during seedling growth (Domonkos et al., 2004; Kobayashi et al., 2016). Besides functioning in oxygenic photosynthesis, PG was also reported to be involved in the phospholipids-galactolipids transition when plant was under phosphate-deprivation condition (Andersson et al., 2003; Frentzen, 2004). Detection of PG content revealed that PG exists in Arabidopsis leaves with kinds of fatty acyl chains, and also exists in oat roots with non-specific fatty acyl chains (Andersson et al., 2003; Frentzen, 2004; Hsu et al., 2007). The PG-target proteins have not been reported in plants. In our study, we found that PG interacted with AtROP6 and stimulated its activity. Further PG content analysis in Arabidopsis revealed that PG existed both in roots and leaves but with different fatty acyl chain patterns, which indicates that PG may have distinct function in the roots from leaves. Taken together, AtROP6 might be one of PG-target proteins in the regulation of cellular activities.

Since AtROP6 acts downstream of auxin to control cell polarity development and cellular activities, we analyzed root PG content with or without NAA treatment using mass spectrometry. The result showed that only C34:3-PG, the major constituent of PG in the root, increased with NAA treatment, while other PG species did not change. However, the link between C34:3-PG and the regulation of AtROP6 activity was unclear in our study, which needs a further experiment to isolate different fatty acyl chain patterns of PG from plant or synthesis them to investigate the fatty acyl chain patterns of PG on the regulation of AtROP6 in the endocytosis process. Since lipids in the cell membrane are distributed asymmetrically (Maekawa and Fairn, 2014), it is also possible that the distribution of PG in the membrane might be involved in the regulation of AtROP6 activity. Interestingly, in bacteria, PG could regulate the activity of *E. coli* GTPase FtsY by binding to its C-terminal GTP-binding domain with the same concentration of PG as we applied on AtROP6. By using Fourier transform infrared (FT-IR) spectroscopy, PG was found to enhance FtsY GTPase activity through changing its conformation (de Leeuw et al., 2000). Whether PG in the plasma membrane interacts with the regulation part of ROP6 to change its conformation or the different conformations exist between ROP6, ROP6^CA^ and ROP6^DN^, which would lead to the variability of ROP6 activity, remains unclear in our study and need further experiments to investigate it.

The ROP GTPase family members serve as critical signal transducers participating in many fundamental cellular activities including cell polarity, cell development, as well as abiotic and biotic stress signaling events (Craddock et al., 2012; Zhang et al., 2014; Lin et al., 2015). In Arabidopsis, AtROP1, AtROP3, and AtROP5 may be involved in the regulation of tip growth of pollen tubes redundantly (Gu et al., 2003; Craddock et al., 2012), wherein, AtROP1 transcript level in pollen is much more abundant than that of AtROP3 and AtROP5, and may play a dominant role in this process and has been extensively studied (Kost et al., 1999; Li et al., 1999, 2008; Gu et al., 2003, 2005, 2006; Hwang et al., 2005, 2010; Zhang and McCormick, 2007; Lee et al., 2008; Wang et al., 2008; Chang et al., 2013; Takeuchi and Higashiyama, 2016; Luo et al., 2017). AtROP3 contributes to polar auxin transport and distribution to control plant patterning and auxin-regulated responses (Huang et al., 2014), and is also activated by RopGEF7 and involved in the regulation of PLETHORA-dependent maintenance of the root stem cell niches (Chen M. et al., 2011). Studies on ROP GTPases in plants also uncover their roles in division plane selection mitotic events (Stockle et al., 2016), biotic stress responses (Venus and Oelmuller, 2013; Zhang et al., 2014), and ABA responses (Craddock et al., 2012). As a molecular switch in cellular activities, ROP GTPases in plants are regulated by many signal molecules such as auxin, ABA, calcium (Li et al., 1999; Yu et al., 2012; Peer, 2013; Miyawaki and Yang, 2014), and by receptor-like kinases such as FERONIA, PRK6 (pollen-specific receptor-like kinase 6) and AtPRK2 (Zhang and McCormick, 2007; Duan et al., 2010; Chang et al., 2013; Takeuchi and Higashiyama, 2016). In this study, besides AtROP6, PG also bound to other ROP protein family members AtROP1 and AtROP3 in the liposome sedimentation assays and MST assay. It is required to further investigate how PG regulates AtROP1 and AtROP3 activity and participates in their cellular functions.

Lipids in plants play important regulatory functions in cellular activities more than just serving as building blocks for membrane structures, and the study to uncover membrane lipid-protein interaction would contribute to the understanding of lipids in the regulation of functional proteins in cellular activities.

## Author Contributions

XH, YY, and YG conceived and designed the research. XH prepared materials, conducted the research, and wrote the original manuscript. YS and GL participated in the preparing materials. YG revised the manuscript. All authors read and approved the final manuscript.

## Conflict of Interest Statement

The authors declare that the research was conducted in the absence of any commercial or financial relationships that could be construed as a potential conflict of interest.

## References

[B1] Andersson M. X. Stridh M. H. Larsson K. E. Liljenberg C. Sandelius A. S. (2003). Phosphate-deficient oat replaces a major portion of the plasma membrane phospholipids with the galactolipid digalactosyldiacylglycerol. *FEBS Lett.* 537 128–132. 10.1016/S0014-5793(03)00109-1 12606044

[B2] Babiychuk E. Müller F. Eubel H. Braun H.-P. Frentzen M. Kushnir S. (2003). *Arabidopsis* phosphatidylglycerophosphate synthase 1 is essential for chloroplast differentiation, but is dispensable for mitochondrial function. *Plant J.* 33 899–909. 10.1046/j.1365-313X.2003.s01680.x 12609031

[B3] Baron C. L. Malhotra V. (2002). Role of diacylglycerol in PKD recruitment to the TGN and protein transport to the plasma membrane. *Science* 295 325–328. 10.1126/science.1066759 11729268

[B4] Basu D. Le J. Zakharova T. Mallery E. L. Szymanski D. B. (2008). A SPIKE1 signaling complex controls actin-dependent cell morphogenesis through the heteromeric WAVE and ARP2/3 complexes. *Proc. Natl. Acad. Sci. U.S.A.* 105 4044–4049. 10.1073/pnas.0710294105 18308939 PMC2268813

[B5] Bloch D. Lavy M. Efrat Y. Efroni I. Bracha-Drori K. Abu-Abied M. (2005). Ectopic expression of an activated RAC in *Arabidopsis* disrupts membrane cycling. *Mol. Biol. Cell* 16 1913–1927. 10.1091/mbc.E04-07-0562 15703216 PMC1073671

[B6] Bogdanov M. Dowhan W. Vitrac H. (2014). Lipids and topological rules governing membrane protein assembly. *Biochim. Biophys. Acta* 1843 1475–1488. 10.1016/j.bbamcr.2013.12.007 24341994 PMC4057987

[B7] Bogdanov M. Xie J. Dowhan W. (2009). Lipid-protein interactions drive membrane protein topogenesis in accordance with the positive inside rule. *J. Biol. Chem.* 284 9637–9641. 10.1074/jbc.R800081200 19074771 PMC2665083

[B8] Chang F. Gu Y. Ma H. Yang Z. (2013). AtPRK2 promotes ROP1 activation via RopGEFs in the control of polarized pollen tube growth. *Mol. Plant* 6 1187–1201. 10.1093/mp/sss103 23024212 PMC3888354

[B9] Chen M. Liu H. Kong J. Yang Y. Zhang N. Li R. (2011). *RopGEF7* regulates PLETHORA-dependent maintenance of the root stem cell niche in *Arabidopsis*. *Plant Cell* 23 2880–2894. 10.1105/tpc.111.085514 21828289 PMC3180798

[B10] Chen X. Grandont L. Li H. Hauschild R. Paque S. Abuzeineh A. (2014). Inhibition of cell expansion by rapid ABP1-mediated auxin effect on microtubules. *Nature* 516 90–93. 10.1038/nature13889 25409144 PMC4257754

[B11] Chen X. Irani N. G. Friml J. (2011). Clathrin-mediated endocytosis: the gateway into plant cells. *Curr. Opin. Plant Biol.* 14 674–682. 10.1016/j.pbi.2011.08.006 21945181

[B12] Chen X. Naramoto S. Robert S. Tejos R. Lofke C. Lin D. (2012). ABP1 and ROP6 GTPase signaling regulate clathrin-mediated endocytosis in *Arabidopsis* roots. *Curr. Biol.* 22 1326–1332. 10.1016/j.cub.2012.05.020 22683261

[B13] Chen Y. H. Du W. Hagemeijer M. C. Takvorian P. M. Pau C. Cali A. (2015). Phosphatidylserine vesicles enable efficient en bloc transmission of enteroviruses. *Cell* 160 619–630. 10.1016/j.cell.2015.01.032 25679758 PMC6704014

[B14] Coursol S. Fan L. M. Le Stunff H. Spiegel S. Gilroy S. Assmann S. M. (2003). Sphingolipid signalling in *Arabidopsis* guard cells involves heterotrimeric G proteins. *Nature* 423 651–654. 10.1038/nature01643 12789341

[B15] Craddock C. Lavagi I. Yang Z. (2012). New insights into Rho signaling from plant ROP/Rac GTPases. *Trends Cell Biol.* 22 492–501. 10.1016/j.tcb.2012.05.002 22795444 PMC3432703

[B16] de Leeuw E. te Kaat K. Moser C. Menestrina G. Demel R. de Kruijff B. (2000). Anionic phospholipids are involved in membrane association of FtsY and stimulate its GTPase activity. *EMBO J.* 19 531–541. 10.1093/emboj/19.4.531 10675322 PMC305591

[B17] Del V. K. Stahelin R. V. (2016). Using surface plasmon resonance to quantitatively assess lipid-protein interactions. *Methods Mol. Biol.* 1376 141–153. 10.1007/978-1-4939-3170-5_12 26552681 PMC5964981

[B18] Dijkman P. M. Watts A. (2015). Lipid modulation of early G protein-coupled receptor signalling events. *Biochim. Biophys. Acta* 1848 2889–2897. 10.1016/j.bbamem.2015.08.004 26275588

[B19] Domonkos I. Malec P. Sallai A. Kovács L. Itoh K. Shen G. (2004). Phosphatidylglycerol is essential for oligomerization of photosystem I reaction center. *Plant Physiol.* 134 1471–1478. 10.1104/pp.103.037754 15064373 PMC419823

[B20] Dong W. Lv H. Xia G. Wang M. (2012). Does diacylglycerol serve as a signaling molecule in plants? *Plant Signal. Behav.* 7 472–475. 10.4161/psb.19644 22499171 PMC3419036

[B21] Dowhan W. (2017). Understanding phospholipid function: Why are there so many lipids? *J. Biol. Chem.* 292 10755–10766. 10.1074/jbc.X117.794891 28490630 PMC5491763

[B22] Dowler S. Kular G. Alessi D. R. (2002). Protein lipid overlay assay. *Sci. STKE* 2002:pl6. 10.1126/stke.2002.129.pl6 11972359

[B23] Drøbak B. K. Watkins P. A. C. Valenta R. Dove S. K. Lloyd C. W. Staiger C. J. (1994). Inhibition of plant plasma membrane phosphoinositide phospholipase C by the actin-binding protein, profilin. *Plant J.* 6 389–400. 10.1046/j.1365-313X.1994.06030389.x

[B24] Duan Q. Kita D. Li C. Cheung A. Y. Wu H. M. (2010). FERONIA receptor-like kinase regulates RHO GTPase signaling of root hair development. *Proc. Natl. Acad. Sci. U.S.A.* 107 17821–17826. 10.1073/pnas.1005366107 20876100 PMC2955125

[B25] Entzian C. Schubert T. (2016). Studying small molecule-aptamer interactions using MicroScale Thermophoresis (MST). *Methods* 97 27–34. 10.1016/j.ymeth.2015.08.023 26334574

[B26] Fan L. Li R. Pan J. Ding Z. Lin J. (2015). Endocytosis and its regulation in plants. *Trends Plant Sci.* 20 388–397. 10.1016/j.tplants.2015.03.014 25914086

[B27] Fang Y. Vilella-Bach M. Bachmann R. Flanigan A. Chen J. (2001). Phosphatidic acid-mediated mitogenic activation of mTOR signaling. *Science* 294 1942–1945. 10.1126/science.1066015 11729323

[B28] Frentzen M. (2004). Phosphatidylglycerol and sulfoquinovosyldiacylglycerol: anionic membrane lipids and phosphate regulation. *Curr. Opin. Plant Biol.* 7 270–276. 10.1016/j.pbi.2004.03.001 15134747

[B29] Fu Y. Xu T. Zhu L. Wen M. Yang Z. (2009). A ROP GTPase signaling pathway controls cortical microtubule ordering and cell expansion in *Arabidopsis*. *Curr. Biol.* 19 1827–1832. 10.1016/j.cub.2009.08.052 19818614 PMC2933814

[B30] Gu Y. Fu Y. Dowd P. Li S. Vernoud V. Gilroy S. (2005). A Rho family GTPase controls actin dynamics and tip growth via two counteracting downstream pathways in pollen tubes. *J. Cell Biol.* 169 127–138. 10.1083/jcb.200409140 15824136 PMC2171904

[B31] Gu Y. Li S. Lord E. M. Yang Z. (2006). Members of a novel class of *Arabidopsis* Rho guanine nucleotide exchange factors control Rho GTPase-dependent polar growth. *Plant Cell* 18 366–381. 10.1105/tpc.105.036434 16415208 PMC1356545

[B32] Gu Y. Vernoud V. Fu Y. Yang Z. (2003). ROP GTPase regulation of pollen tube growth through the dynamics of tip-localized F-actin. *J. Exp. Bot.* 54 93–101. 10.1093/jxb/erg035 12456759

[B33] Heilmann I. (2016a). Phosphoinositide signaling in plant development. *Development* 143 2044–2055. 10.1242/dev.136432 27302395

[B34] Heilmann I. (2016b). Plant phosphoinositide signaling - dynamics on demand. *Biochim. Biophys. Acta* 1861 1345–1351. 10.1016/j.bbalip.2016.02.013 26924252

[B35] Hsu F.-F. Turk J. Williams T. D. Welti R. (2007). Electrospray ionization multiple stage quadrupole ion-trap and tandem quadrupole mass spectrometric studies on phosphatidylglycerol from Arabidopsis leaves. *J. Am. Soc. Mass Spectr.* 18 783–790. 10.1016/j.jasms.2006.12.012 17303435 PMC2747347

[B36] Huang J. B. Liu H. Chen M. Li X. Wang M. Yang Y. (2014). ROP3 GTPase contributes to polar auxin transport and auxin responses and is important for embryogenesis and seedling growth in *Arabidopsis*. *Plant Cell* 26 3501–3518. 10.1105/tpc.114.127902 25217509 PMC4213153

[B37] Hwang J. U. Gu Y. Lee Y. J. Yang Z. (2005). Oscillatory ROP GTPase activation leads the oscillatory polarized growth of pollen tubes. *Mol. Biol. Cell* 16 5385–5399. 10.1091/mbc.E05-05-0409 16148045 PMC1266434

[B38] Hwang J. U. Wu G. Yan A. Lee Y. J. Grierson C. S. Yang Z. (2010). Pollen-tube tip growth requires a balance of lateral propagation and global inhibition of Rho-family GTPase activity. *J. Cell Sci.* 123 340–350. 10.1242/jcs.039180 20053639 PMC2816183

[B39] Irani N. G. Russinova E. (2009). Receptor endocytosis and signaling in plants. *Curr. Opin. Plant Biol.* 12 653–659. 10.1016/j.pbi.2009.09.011 19850509

[B40] Jouhet J. Marechal E. Block M. A. (2007). Glycerolipid transfer for the building of membranes in plant cells. *Prog. Lipid Res.* 46 37–55. 10.1016/j.plipres.2006.06.002 16970991

[B41] Ke D. Fang Q. Chen C. Zhu H. Chen T. Chang X. (2012). The small GTPase ROP6 interacts with NFR5 and is involved in nodule formation in *Lotus japonicus*. *Plant Physiol.* 159 131–143. 10.1104/pp.112.197269 22434040 PMC3375957

[B42] Kim H. J. Jeong M. H. Kim K. R. Jung C. Y. Lee S. Y. Kim H. (2016). Protein arginine methylation facilitates KCNQ channel-PIP2 interaction leading to seizure suppression. *eLife* 5:e17159. 10.7554/eLife.17159 27466704 PMC4996652

[B43] Kim Y. J. Kim J. E. Lee J. H. Lee M. H. Jung H. W. Bahk Y. Y. (2004). The Vr-PLC3 gene encodes a putative plasma membrane-localized phosphoinositide-specific phospholipase C whose expression is induced by abiotic stress in mung bean (*Vigna radiata* L.). *FEBS Lett.* 556 127–136. 10.1016/S0014-5793(03)01388-7 14706839

[B44] Kobayashi K. Endo K. Wada H. (2016). Multiple impacts of loss of plastidic phosphatidylglycerol biosynthesis on photosynthesis during seedling growth of Arabidopsis. *Front. Plant Sci.* 7:336. 10.3389/fpls.2016.00336 27047516 PMC4800280

[B45] Kost B. Lemichez E. Spielhofer P. Hong Y. Tolias K. Carpenter C. (1999). Rac homologues and compartmentalized phosphatidylinositol 4, 5-bisphosphate act in a common pathway to regulate polar pollen tube growth. *J. Cell Biol.* 145 317–330. 10.1083/jcb.145.2.317 10209027 PMC2133117

[B46] Lam S. M. Shui G. H. (2013). Lipidomics as a principal tool for advancing biomedical research. *J. Genet. Genomics* 40 375–390. 10.1016/j.jgg.2013.06.007 23969247

[B47] Lee Y. J. Szumlanski A. Nielsen E. Yang Z. (2008). Rho-GTPase–dependent filamentous actin dynamics coordinate vesicle targeting and exocytosis during tip growth. *J. Cell Biol.* 181 1155–1168. 10.1083/jcb.200801086 18591430 PMC2442199

[B48] Leeuwen W. V. Okrész L. Bögre L. Munnik T. (2004). Learning the lipid language of plant signalling. *Trends Plant Sci.* 9 378–384. 10.1016/j.tplants.2004.06.008 15358268

[B49] Lemmon M. A. (2003). Phosphoinositide recognition domains. *Traffic* 4 201–213. 10.1034/j.1600-0854.2004.00071.x 12694559

[B50] Li H. Lin Y. Heath R. M. Zhu M. X. Yang Z. (1999). Control of pollen tube tip growth by a Rop GTPase-dependent pathway that leads to tip-localized calcium influx. *Plant Cell* 11 1731–1742. 10488239 10.1105/tpc.11.9.1731 PMC144310

[B51] Li H. Wu G. Ware D. Davis K. R. Yang Z. (1998). Arabidopsis Rho-related GTPases: differential gene expression in pollen and polar localization in fission yeast. *Plant Physiol.* 118 407–417. 10.1104/pp.118.2.407 9765526 PMC34816

[B52] Li N. Xu C. Li-Beisson Y. Philippar K. (2016). Fatty acid and lipid transport in plant cells. *Trends Plant Sci.* 21 145–158. 10.1016/j.tplants.2015.10.011 26616197

[B53] Li S. Gu Y. Yan A. Lord E. Yang Z. B. (2008). RIP1 (ROP Interactive Partner 1)/ICR1 marks pollen germination sites and may act in the ROP1 pathway in the control of polarized pollen growth. *Mol. Plant* 1 1021–1035. 10.1093/mp/ssn051 19825600 PMC9345201

[B54] Li Y. Beisson F. Ohlrogge J. Pollard M. (2007). Monoacylglycerols are components of root waxes and can be produced in the aerial cuticle by ectopic expression of a suberin-associated acyltransferase. *Plant Physiol.* 144 1267–1277. 10.1104/pp.107.099432 17496107 PMC1914122

[B55] Lin C. C. Melo F. A. Ghosh R. Suen K. M. Stagg L. J. Kirkpatrick J. (2012). Inhibition of basal FGF receptor signaling by dimeric Grb2. *Cell* 149 1514–1524. 10.1016/j.cell.2012.04.033 22726438

[B56] Lin D. Cao L. Zhou Z. Zhu L. Ehrhardt D. Yang Z. (2013). Rho GTPase signaling activates microtubule severing to promote microtubule ordering in *Arabidopsis*. *Curr. Biol.* 23 290–297. 10.1016/j.cub.2013.01.022 23394835

[B57] Lin D. Nagawa S. Chen J. Cao L. Chen X. Xu T. (2012). A ROP GTPase-dependent auxin signaling pathway regulates the subcellular distribution of PIN2 in *Arabidopsis* roots. *Curr. Biol.* 22 1319–1325. 10.1016/j.cub.2012.05.019 22683260 PMC3407329

[B58] Lin D. Ren H. Fu Y. (2015). ROP GTPase-mediated auxin signaling regulates pavement cell interdigitation in *Arabidopsis thaliana*. *J. Integr. Plant Biol.* 57 31–39. 10.1111/jipb.12281 25168157

[B59] Luo N. Yan A. Liu G. Guo J. Rong D. Kanaoka M. M. (2017). Exocytosis-coordinated mechanisms for tip growth underlie pollen tube growth guidance. *Nat. Commun.* 8:1687. 10.1038/s41467-017-01452-0 29162819 PMC5698331

[B60] Maekawa M. Fairn G. D. (2014). Molecular probes to visualize the location, organization and dynamics of lipids. *J. Cell Sci.* 127 4801–4812. 10.1242/jcs.150524 25179600

[B61] Mandal M. K. Chandra-Shekara A. C. Jeong R. D. Yu K. Zhu S. Chanda B. (2012). Oleic acid-dependent modulation of NITRIC OXIDE ASSOCIATED1 protein levels regulates nitric oxide-mediated defense signaling in *Arabidopsis*. *Plant Cell* 24 1654–1674. 10.1105/tpc.112.096768 22492810 PMC3398570

[B62] Manoharan K. Prasad R. Guha-Mukherjee S. (1985). Greening-related lipid changes in leaves, protoplasts and a plasmamembrane-enriched fraction of pea. *Phytochemistry* 24 431–433. 10.1016/S0031-9422(00)80741-4

[B63] Milne S. B. Ivanova P. T. DeCamp D. Hsueh R. C. Brown H. A. (2005). A targeted mass spectrometric analysis of phosphatidylinositol phosphate species. *J. Lipid Res.* 46 1796–1802. 10.1194/jlr.D500010-JLR200 15897608

[B64] Miyawaki K. N. Yang Z. (2014). Extracellular signals and receptor-like kinases regulating ROP GTPases in plants. *Front. Plant Sci.* 5:449. 10.3389/fpls.2014.00449 25295042 PMC4170102

[B65] Moreau P. Bessoule J. J. Mongrand S. Testet E. Vincent P. Cassagne C. (1998). Lipid trafficking in plant cells. *Prog. Lipid Res.* 37 371–391. 10.1016/S0163-7827(98)00016-2 10209654

[B66] Munnik T. (2001). Phosphatidic acid: an emerging plant lipid second messenger. *Trends Plant Sci.* 6 227–233. 10.1016/S1360-1385(01)01918-5 11335176

[B67] Munnik T. Wierzchowiecka M. (2013). Lipid-binding analysis using a fat blot assay. *Methods Mol. Biol.* 1009 253–259. 10.1007/978-1-62703-401-2_23 23681540

[B68] Nagawa S. Xu T. Lin D. Dhonukshe P. Zhang X. Friml J. (2012). ROP GTPase-dependent actin microfilaments promote PIN1 polarization by localized inhibition of clathrin-dependent endocytosis. *PLoS Biol.* 10:e1001299. 10.1371/journal.pbio.1001299 22509133 PMC3317906

[B69] Ng C. K. Carr K. McAinsh M. R. Powell B. Hetherington A. M. (2001). Drought-induced guard cell signal transduction involves sphingosine-1-phosphate. *Nature* 410 596–599. 10.1038/35069092 11279499

[B70] Paciorek T. Zazimalova E. Ruthardt N. Petrasek J. Stierhof Y. D. Kleine-Vehn J. (2005). Auxin inhibits endocytosis and promotes its own efflux from cells. *Nature* 435 1251–1256. 10.1038/nature03633 15988527

[B71] Pan J. Fujioka S. Peng J. Chen J. Li G. Chen R. (2009). The E3 ubiquitin ligase SCFTIR1/AFB and membrane sterols play key roles in auxin regulation of endocytosis, recycling, and plasma membrane accumulation of the auxin efflux transporter PIN2 in *Arabidopsis thaliana*. *Plant Cell* 21 568–580. 10.1105/tpc.108.061465 19218398 PMC2660622

[B72] Peer W. A. (2013). From perception to attenuation: auxin signalling and responses. *Curr. Opin. Plant Biol.* 16 561–568. 10.1016/j.pbi.2013.08.003 24004572

[B73] Poraty-Gavra L. Zimmermann P. Haigis S. Bednarek P. Hazak O. Stelmakh O. R. (2013). The Arabidopsis Rho of plants GTPase AtROP6 functions in developmental and pathogen response pathways. *Plant Physiol.* 161 1172–1188. 10.1104/pp.112.213165 23319551 PMC3585588

[B74] Ren H. Dang X. Yang Y. Huang D. Liu M. Gao X. (2016). SPIKE1 activates ROP GTPase to modulate petal growth and shape. *Plant Physiol.* 172 358–371. 10.1104/pp.16.00788 27440754 PMC5074625

[B75] Rigal A. Doyle S. M. Robert S. (2015). Live cell imaging of FM4-64, a tool for tracing the endocytic pathways in *Arabidopsis* root cells. *Methods Mol. Biol.* 1242 93–103. 10.1007/978-1-4939-1902-4_9 25408447

[B76] Ritter D. Yopp J. H. (1993). Plasma membrane lipid composition of the halophilic cyanobacterium *Aphanothece halophytica*. *Arch. Microbiol.* 159 435–439. 10.1007/BF00288590

[B77] Sheen J. (2001). Signal transduction in maize and Arabidopsis mesophyll protoplasts. *Plant Physiol.* 127 1466–1475. 10.1104/pp.010820 11743090 PMC1540179

[B78] Simon M. L. Platre M. P. Assil S. van Wijk R. Chen W. Y. Chory J. (2014). A multi-colour/multi-affinity marker set to visualize phosphoinositide dynamics in Arabidopsis. *Plant J.* 77 322–337. 10.1111/tpj.12358 24147788 PMC3981938

[B79] Sorek N. Segev O. Gutman O. Bar E. Richter S. Poraty L. (2010). An S-acylation switch of conserved G domain cysteines is required for polarity signaling by ROP GTPases. *Curr. Biol.* 20 914–920. 10.1016/j.cub.2010.03.057 20451389

[B80] Stockle D. Herrmann A. Lipka E. Lauster T. Gavidia R. Zimmermann S. (2016). Putative RopGAPs impact division plane selection and interact with kinesin-12 POK1. *Nat. Plants* 2:16120. 10.1038/nplants.2016.120 27501519

[B81] Suh B. C. Hille B. (2008). PIP2 is a necessary cofactor for ion channel function: how and why? *Annu. Rev. Biophys.* 37 175–195. 10.1146/annurev.biophys.37.032807.125859 18573078 PMC2692585

[B82] Sun F. Kale S. D. Azurmendi H. F. Li D. Tyler B. M. Capelluto D. G. (2013). Structural basis for interactions of the *Phytophthora sojae* RxLR effector Avh5 with phosphatidylinositol 3-phosphate and for host cell entry. *Mol. Plant Microbe Interact.* 26 330–344. 10.1094/MPMI-07-12-0184-R 23075041

[B83] Swamy M. J. Sankhala R. S. (2013). *Probing the Thermodynamics of Protein-lipid Interactions by Isothermal Titration Calorimetry.* New York City, NY: Humana Press. 10.1007/978-1-62703-275-9_3 23404271

[B84] Takeuchi H. Higashiyama T. (2016). Tip-localized receptors control pollen tube growth and LURE sensing in *Arabidopsis*. *Nature* 531 245–248. 10.1038/nature17413 26961657

[B85] Tejos R. Sauer M. Vanneste S. Palacios-Gomez M. Li H. Heilmann M. (2014). Bipolar plasma membrane distribution of phosphoinositides and their requirement for auxin-mediated cell polarity and patterning in *Arabidopsis*. *Plant Cell* 26 2114–2128. 10.1105/tpc.114.126185 24876254 PMC4079372

[B86] Venus Y. Oelmuller R. (2013). *Arabidopsis* ROP1 and ROP6 influence germination time, root morphology, the formation of F-actin bundles, and symbiotic fungal interactions. *Mol. Plant* 6 872–886. 10.1093/mp/sss101 23118477

[B87] Wang C. Hu T. Yan X. Meng T. Wang Y. Wang Q. (2016). Differential regulation of clathrin and its adaptor proteins during membrane recruitment for endocytosis. *Plant Physiol.* 171 215–229. 10.1104/pp.15.01716 26945051 PMC4854679

[B88] Wang C. Xu X. Hong Z. Feng Y. Zhang Z. (2015a). Involvement of ROP6 and clathrin in nodulation factor signaling. *Plant Signal. Behav.* 10:e1033127. 10.1080/15592324.2015.1033127 26251877 PMC4622583

[B89] Wang C. Yan X. Chen Q. Jiang N. Fu W. Ma B. (2013). Clathrin light chains regulate clathrin-mediated trafficking, auxin signaling, and development in *Arabidopsis*. *Plant Cell* 25 499–516. 10.1105/tpc.112.108373 23424247 PMC3608774

[B90] Wang C. Zhu M. Duan L. Yu H. Chang X. Li L. (2015b). *Lotus japonicus* clathrin heavy Chain1 is associated with Rho-Like GTPase ROP6 and involved in nodule formation. *Plant Physiol.* 167 1497–1510. 10.1104/pp.114.256107 25717037 PMC4378172

[B91] Wang Y. Zhang W. Z. Song L. F. Zou J. J. Su Z. Wu W. H. (2008). Transcriptome analyses show changes in gene expression to accompany pollen germination and tube growth in Arabidopsis. *Plant Physiol.* 148 1201–1211. 10.1104/pp.108.126375 18775970 PMC2577266

[B92] Xu T. (2012). Rho GTPase activity analysis in plant cells. *Methods Mol. Biol.* 876 135–144. 10.1007/978-1-61779-809-2_10 22576091

[B93] Xu T. Wen M. Nagawa S. Fu Y. Chen J. G. Wu M. J. (2010). Cell surface- and rho GTPase-based auxin signaling controls cellular interdigitation in Arabidopsis. *Cell* 143 99–110. 10.1016/j.cell.2010.09.003 20887895 PMC2950838

[B94] Yang W. Pollard M. Li-Beisson Y. Beisson F. Feig M. Ohlrogge J. (2010). A distinct type of glycerol-3-phosphate acyltransferase with sn-2 preference and phosphatase activity producing 2-monoacylglycerol. *Proc. Natl. Acad. Sci. U.S.A.* 107 12040–12045. 10.1073/pnas.0914149107 20551224 PMC2900678

[B95] Young B. P. Shin J. J. Orij R. Chao J. T. Li S. C. Guan X. L. (2010). Phosphatidic acid is a pH biosensor that links membrane biogenesis to metabolism. *Science* 329 1085–1088. 10.1126/science.1191026 20798321

[B96] Yu F. Qian L. Nibau C. Duan Q. Kita D. Levasseur K. (2012). FERONIA receptor kinase pathway suppresses abscisic acid signaling in *Arabidopsis* by activating ABI2 phosphatase. *Proc. Natl. Acad. Sci. U.S.A.* 109 14693–14698. 10.1073/pnas.1212547109 22908257 PMC3437822

[B97] Yu L. Nie J. Cao C. Jin Y. Yan M. Wang F. (2010). Phosphatidic acid mediates salt stress response by regulation of MPK6 in *Arabidopsis thaliana*. *New Phytol.* 188 762–773. 10.1111/j.1469-8137.2010.03422.x 20796215

[B98] Yuan X. Li Y. Liu S. Xia F. Li X. Qi B. (2014). Accumulation of eicosapolyenoic acids enhances sensitivity to abscisic acid and mitigates the effects of drought in transgenic *Arabidopsis thaliana*. *J. Exp. Bot.* 65 1637–1649. 10.1093/jxb/eru031 24609499 PMC3967093

[B99] Zhang Q. Lin F. Mao T. Nie J. Yan M. Yuan M. (2012). Phosphatidic acid regulates microtubule organization by interacting with MAP65-1 in response to salt stress in *Arabidopsis*. *Plant Cell* 24 4555–4576. 10.1105/tpc.112.104182 23150630 PMC3531852

[B100] Zhang W. Qin C. Zhao J. Wang X. (2004). Phospholipase D alpha 1-derived phosphatidic acid interacts with ABI1 phosphatase 2C and regulates abscisic acid signaling. *Proc. Natl. Acad. Sci. U.S.A.* 101 9508–9513. 10.1073/pnas.0402112101 15197253 PMC439007

[B101] Zhang Y. McCormick S. (2007). A distinct mechanism regulating a pollen-specific guanine nucleotide exchange factor for the small GTPase Rop in *Arabidopsis thaliana*. *Proc. Natl. Acad. Sci. U.S.A.* 104 18830–18835. 10.1073/pnas.0705874104 18000057 PMC2141862

[B102] Zhang Z. Yang F. Na R. Zhang X. Yang S. Gao J. (2014). AtROP1 negatively regulates potato resistance to *Phytophthora infestans* via NADPH oxidase-mediated accumulation of H_2_O_2_. *BMC Plant Biol.* 14:392. 10.1186/s12870-014-0392-2 25547733 PMC4323192

[B103] Zhao S. Jiang Y. Zhao Y. Huang S. Yuan M. Zhao Y. (2016). CASEIN KINASE1-LIKE PROTEIN2 regulates actin filament stability and stomatal closure via phosphorylation of actin depolymerizing factor. *Plant Cell* 28 1422–1439. 10.1105/tpc.16.00078 27268429 PMC4944410

