# Supplementary material for: Activation of ROP6 GTPase by Phosphatidylglycerol in Arabidopsis
Source: Front Plant Sci. 2018 Mar 15;9:347. doi: 10.3389/fpls.2018.00347 (PMC5862815; doi:10.3389/fpls.2018.00347)
Supplement: Supplementary file 1 [file Data_Sheet_1.DOCX]

Supplemental Figure 1. PG binds to GST-AtROP1 and GST-AtROP3.


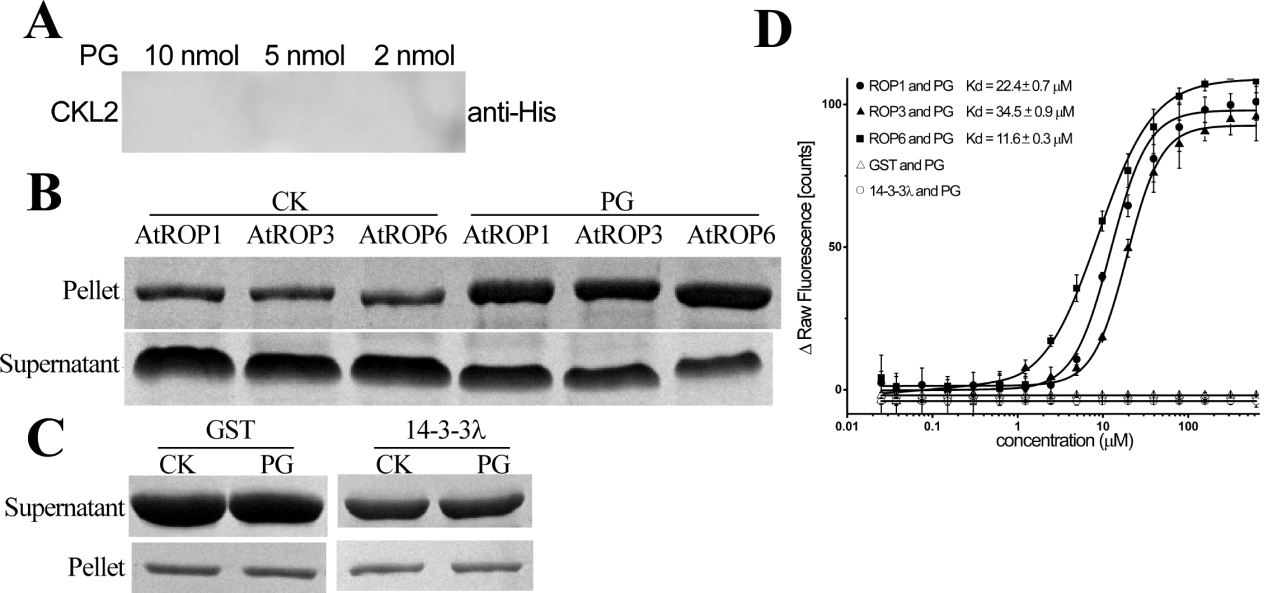


(A) Lipid–protein overlay assay between recombinant protein His-CKL2 and PG. PG-bound CKL2 was detected by immunoblot using anti-His antibodies.

(B) Liposome sedimentation assay between the recombinant proteins GST-AtROP1, GST-AtROP3, GST-AtROP6 and liposomes with or without PG. CK Liposome mixtures were prepared by 200 µg DOPC/DOPE (1:1), PG Liposome mixtures were prepared by 160 µg DOPC/DOPE (1:1) added with 40 µg PG. Liposomes were incubated with proteins GST-AtROP1, GST-AtROP3 and GST-AtROP6 separately, and centrifuged to get liposome-bound pellet protein fractions and supernatant protein fractions. Further detection was performed by SDS-PAGE and Coomassie Blue Staining.

(C) Liposome sedimentation assay between the recombinant proteins GST tag, GST-14-3-3λ and liposomes with or without PG. CK Liposome mixtures were prepared by 200 µg DOPC/DOPE (1:1), PG Liposome mixtures were prepared by 160 µg DOPC/DOPE (1:1) added with 40 µg PG. Liposomes were incubated with proteins GST tag and GST-14-3-3λ separately, and centrifuged to get liposome-bound pellet protein fractions and supernatant protein fractions. Further detection was performed by SDS-PAGE and Coomassie Blue Staining.

(D) Microscale thermophoresis assay between the recombinant proteins GST tag, GST-AtROP1, GST-AtROP3, GST-AtROP6, GST-14-3-3λ and PG. Proteins were labeled with NHS NT-647 dye and kept at a constant concentration (100 nM). PG was hydrated in 1 mL PBST buffer (0.005% tween 20, pH = 7.5) to get the stock solution 1 mg/mL. PG was titrated from 30 nM to 300 μM and the assay was carried out with 20% LED power and 20% MST power.

The experiment was performed at least three independent times.

Supplemental Figure 2. PG binds to GST-AtROP6^CA^ and GST-AtROP6^DN^.


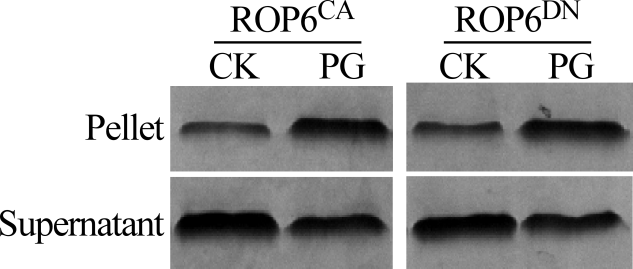


Liposome sedimentation assay between the recombinant proteins GST-ROP6^CA^, GST-ROP6^DN^ and liposomes with or without PG. CK Liposome mixtures were prepared by 200 µg DOPC/DOPE (1:1), PG Liposome mixtures were prepared by 160 µg DOPC/DOPE (1:1) added with 40 µg PG. Liposomes were incubated with proteins GST-ROP6^CA^ and GST-ROP6^DN^ separately, and centrifuged to get liposome-bound pellet protein fractions and supernatant protein fractions. Further detection was performed by SDS-PAGE and Coomassie Blue Staining.

The experiment was performed at least three independent times.

Supplemental Figure 3. PC and PE do not activate ROP6.


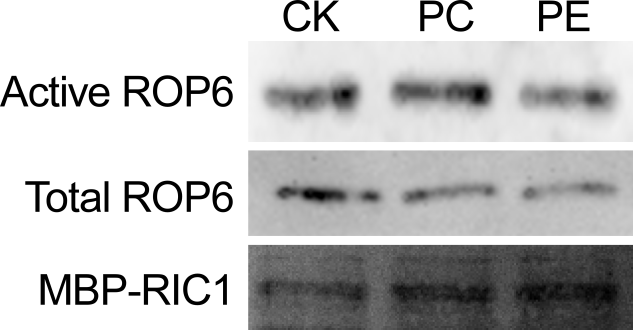


Immunoblot analysis of active ROP6 and total ROP6 from *35S::GFP-ROP6* seedlings treated with 20 µM PC or PE. The upper lane shows the immunoblot analysis of active ROP6, which was from MBP-RIC1 bound fraction. The middle lane shows the immunoblot analysis of total ROP6, which was prepared from total protein extraction. ROP6 was detected by immunoblot using anti-GFP antibody. The lower lane shows the Coomassie Brilliant Blue staining of MBP-RIC1 on PVDF membrane after immunoblot analysis.

The experiment was performed at least three independent times.

Supplemental Figure 4. PC and PE do not regulate ROP6-mediated endocytosis regulation.


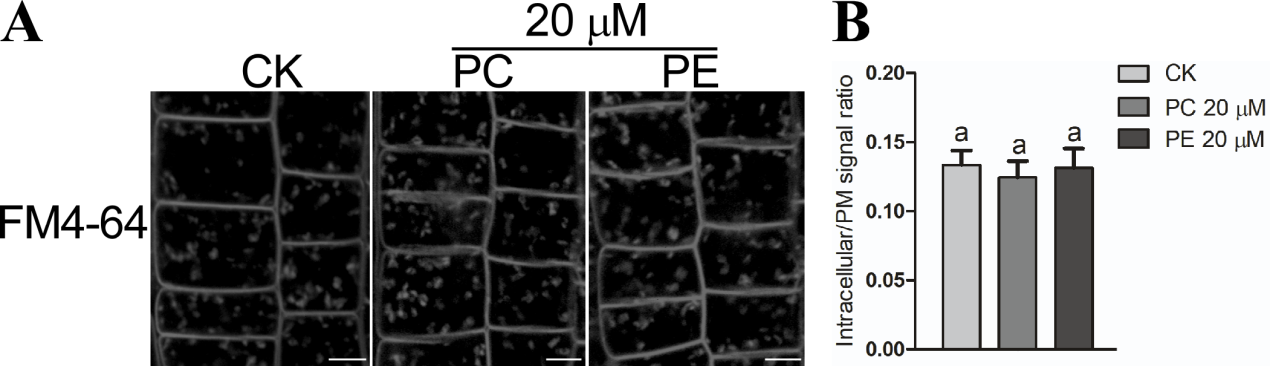


(A) FM4-64 uptake in the seedlings of Col-0 treated with 20 μM PC or PE. The seedlings were stained with FM4-64 and endocytosis process was observed using confocal microscopy. Scale bar represents 5 μm.

(B) Ratio quantitation analysis of FM4-64 signal in panel A. The FM4-64 signal in the cytoplasm and the plasma membrane was performed by a separate measurement using ImageJ software, and then the ratio was calculated (n > 20).

The bar represents the mean and the error bar represents the standard error. The data were calculated from at least three independent experiments. The statistical significance was analyzed by a Student’s t-test and no significant difference (P > 0.05) was indicated by the same lowercase letters.

Supplemental Figure 5. PIN2-GFP accumulation in BFA bodies was still observed in the presence of CHX.


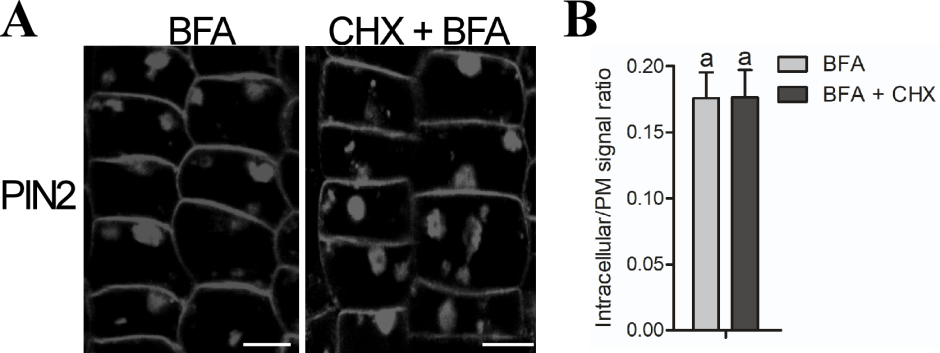


(A) BFA-induced PIN2 internalization with CHX treatment. The seedlings were treated with or without CHX for 30 min, followed by treatment with CHX plus BFA or BFA for 2 hours. The concentration of the chemicals used: CHX, 50 μM; BFA, 50 μM. Scale bar represents 5 μm.

(B) Ratio quantitation analysis of PIN2-GFP signal in panel A. The PIN2-GFP signal in the cytoplasm and the plasma membrane was performed by a separate measurement using ImageJ software, and then the ratio was calculated (n > 20).

The bar represents the mean and the error bar represents the standard error. The data were calculated from at least three independent experiments. The statistical significance was analyzed by a Student’s t-test and no significant difference (P > 0.05) was indicated by the same lowercase letters.

Supplemental Figure 6. PC and PE do not inhibit BFA-induced PIN2 internalization.


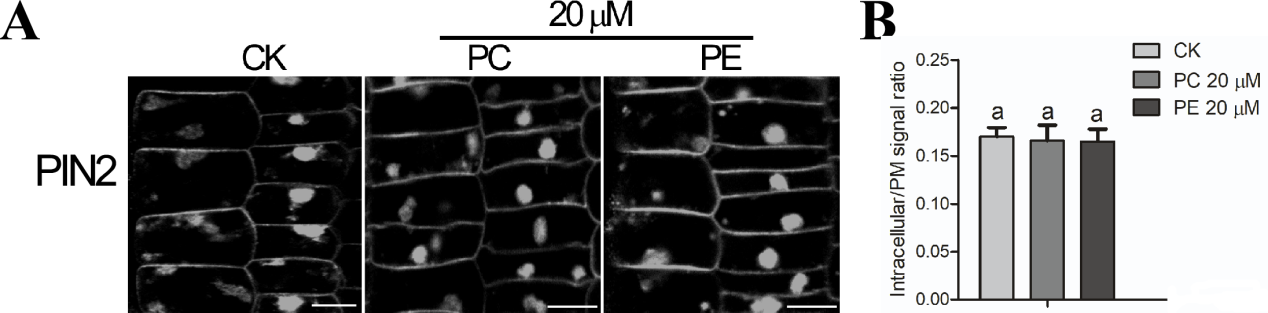


(A) BFA-induced PIN2 internalization after PC and PE treatment. The seedlings were treated with or without 20 μM PC or PE, and then co-treated with 50 μM BFA for 2 hours, and BFA bodies were observed. Scale bar represents 5 μm.

(B) Ratio quantitation analysis of PIN2-GFP signal in panel A. The PIN2-GFP signal in the cytoplasm and the plasma membrane was performed by a separate measurement using ImageJ software, and then the ratio was calculated (n > 20).

The bar represents the mean and the error bar represents the standard error. The data were calculated from at least three independent experiments. The statistical significance was analyzed by a Student’s t-test and no significant difference (P > 0.05) was indicated by the same lowercase letters.

Supplemental Figure 7. PG does not influence exocytosis.


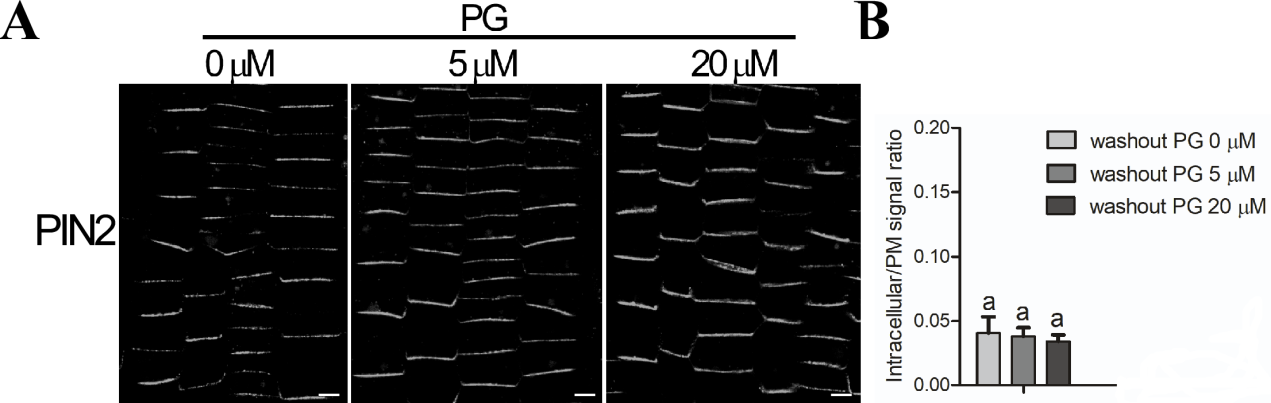


(A) BFA-induced PIN2 internalization disappeared after washout experiment. The seedlings (*PIN2-GFP* in Col-0 background) were treated with indicated amounts of PG, and then co-treated with 50 μM BFA for 2 hours. The seedlings were then applied washout for 1 hour and PIN2-GFP signal was observed. Scale bar represents 5 μm.

(B) Ratio quantitation analysis of PIN2-GFP signal in panel A. The PIN2-GFP signal in the cytoplasm and the plasma membrane was performed by a separate measurement using ImageJ software, and then the ratio was calculated (n > 20).

The bar represents the mean and the error bar represents the standard error. The data were calculated from at least three independent experiments. The statistical significance was analyzed by a Student’s t-test and no significant difference (P > 0.05) was indicated by the same lowercase letters.

Supplemental Figure 8. Application of PG at 50 μM shows a waved pattern on root growth.





Seedlings of Col-0 were grown on MS medium for 4 days and then transferred to MS medium supplemented with or without PG. After growing vertically for 3 days, the pictures were taken. The arrow on the right represents the point that the seedlings were transferred from MS medium plate.

Supplemental Figure 9. PC and PE do not regulate gravitropic response in the seedlings of Col-0.


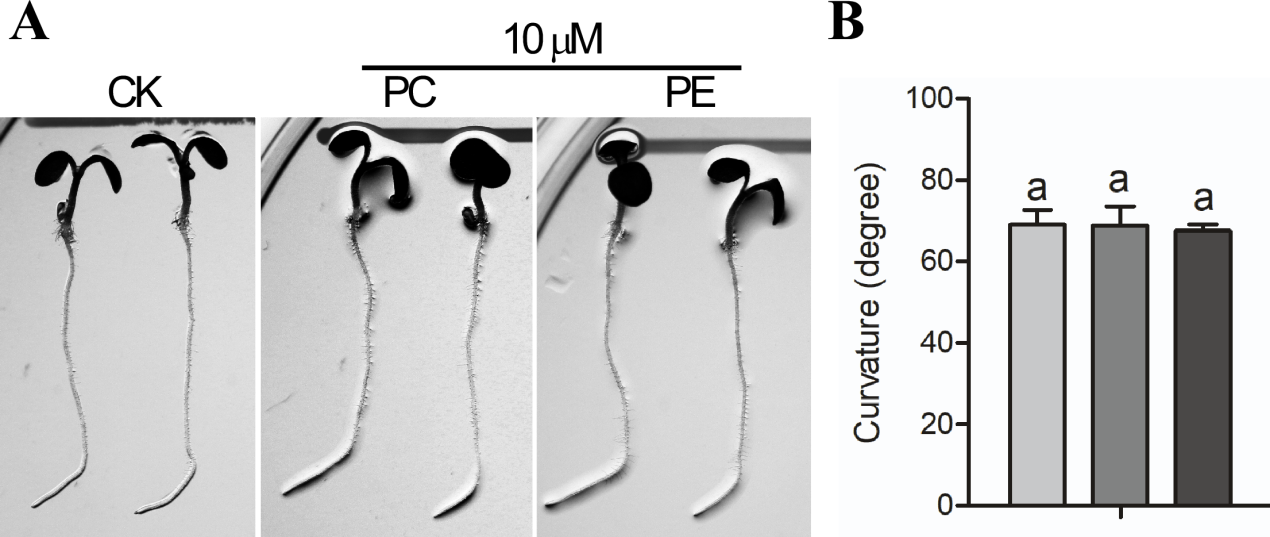


(A) Gravitropic response in the seedlings of Col-0 with the treatment of 10 μM PC or PE. Seedlings of Col-0 were treated with 10 μM PC or PE for 12 hours, and then rotated 90° for gravistimulation.

(B) Root tropic bending curvatures of the seedlings at 12 hours after reorientation in panel A.

The bar represents the mean and the error bar represents the standard error. The data were calculated from at least three independent experiments. The statistical significance was analyzed by a Student’s t-test and no significant difference (P > 0.05) was indicated by the same lowercase letters.

Supplemental Figure 10. PG does not enhance root growth in the seedlings of Col-0.


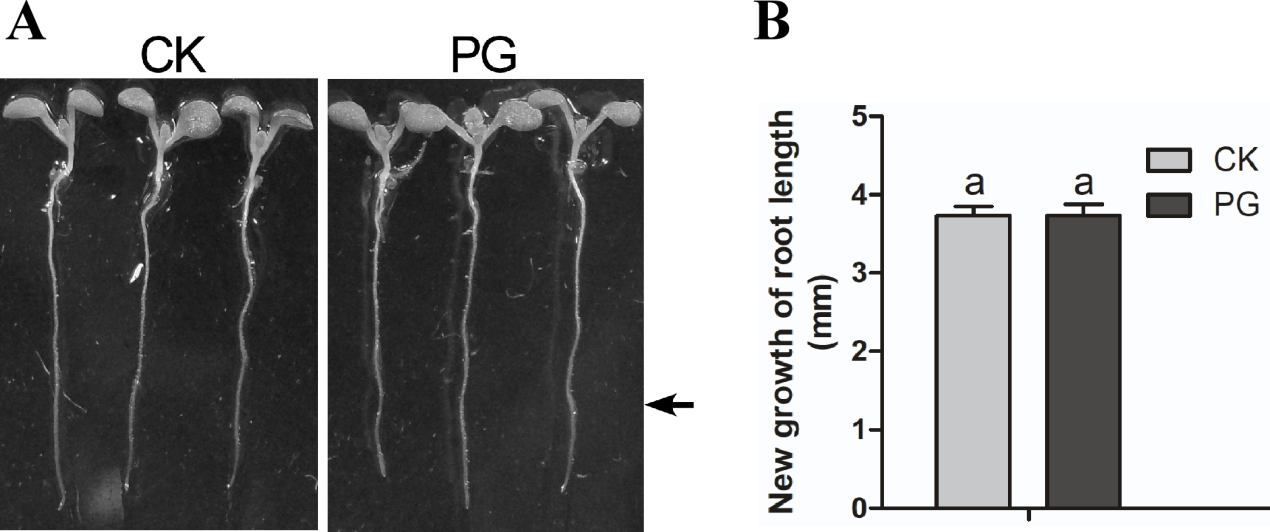


(A) Seedlings of Col-0 were grown on MS medium for 4 days and then transferred to MS medium supplemented with or without 10 μM PG. The arrow on the right represents the point that the seedlings were transferred from MS medium plate.

(B) The root length from the transferred point in panel A.

The bar represents the mean and the error bar represents the standard error. The data were calculated from at least three independent experiments. The statistical significance was analyzed by a Student’s t-test and no significant difference (P > 0.05) was indicated by the same lowercase letters.

Supplemental Table 1. Primers used for plasmid construction

| Primer name | Sequence |
| --- | --- |
| R1-Ef | CGGAATTCATGAGCGCTTCGAGGTTC |
| R1-Sr | GCGTCGACTCATAGAATGGAGCATGC |
| R3-Ef | CGGAATTCATGAGCGCTTCGAGGTTC |
| R3-Sr | GCGTCGACTTACAAAATGGAGCAGGC |
| R6-Bf | CGGGATCCATGAGTGCTTCAAGGTTT |
| R6-Er | CGGAATTCTCAGAGTATAGAACAACC |
| R6-Hr | CCAAGCTTTCAGAGTATAGAACAACC |
| λ-Bf | GCGGATCCATGGCGGCGACATTAGGCAGAG |
| λ-Sr | GTACGTCGACTCAGGCCTCGTCCATCTGCTC |
| R6GV-f | AAGTGTGTCACTGTCGGCGACGTTGCTGTTGGA |
| R6GV-r | AAGACAAGTCTTTCCAACAGCAACGTCGCCGAC |
| R6TN-f | GGCGACGGTGCTGTTGGAAAGAATTGTCTTCTC |
| R6TN-r | GTGTAGGAGATGAGAAGACAATTCTTTCCAAC |
